# A novel MADM algorithm for landfill site selection based on q-rung orthopair probabilistic hesitant fuzzy power Muirhead mean operator

**DOI:** 10.1371/journal.pone.0258448

**Published:** 2021-10-21

**Authors:** Yaojun Ren, Xiujiu Yuan, Ruojing Lin

**Affiliations:** 1 Department of Basic Sciences, Air Force Engineering University, Xi’an, Shaanxi Province, China; 2 College of Geographical Sciences, Shanxi Normal University, Linfen, Shanxi Province, China; University of Defence in Belgrade, SERBIA

## Abstract

With the rapid development of economy and the acceleration of urbanization, the garbage produced by urban residents also increases with the increase of population. In many big cities, the phenomenon of "garbage siege" has seriously affected the development of cities and the lives of residents. Sanitary landfill is an important way of municipal solid waste disposal. However, due to the restriction of social, environmental and economic conditions, landfill site selection has become a very challenging task. In addition, landfill site selection is full of uncertainty and complexity due to the lack of cognitive ability of decision-makers and the existence of uncertain information in the decision-making process. Therefore, a novel multi-attribute decision making method based on q-rung orthopair probabilistic hesitant fuzzy power weight Muirhead mean operator is proposed in this paper, which can solve the problem of landfill site selection well. This method uses probability to represent the hesitance of decision maker and retains decision information more comprehensively. The negative effect of abnormal data on the decision result is eliminated by using the power average operator. Muirhead mean operator is used to describe the correlation between attributes. Then, an example of landfill site selection is given to verify the effectiveness of the proposed method, and the advantages of the proposed method are illustrated by parameter analysis and comparative analysis. The results show that this method has a wider space for information expression, gives the decision maker a great degree of freedom in decision-making, and has robustness.

## 1. Introduction

In recent years, with the rapid economic development and the acceleration of urbanization, the urban population has gradually increased, and the generated waste has also become more, such as household waste, construction waste, and industrial waste. In many large cities, the phenomenon of "garbage siege" has become more and more intense, and the hazard and disposal of garbage has become an important social issue affecting urban development [1]. Since the beginning of the 21^st^ century, the average annual growth rate of global waste has reached 8.42%, while in developing countries, such as China and India, this number has even exceeded 10% [2]. Waste management includes waste reduction, reuse, material recycling, energy recovery, incineration and landfill, etc [3]. We are committed to reducing waste generation from the source, recycling and recovery aspects, but the amount of waste generated in cities every day is still huge. At present, the main treatment methods of municipal solid waste are sanitary landfill, composting and incineration for power generation [4]. Considering the recycling of waste, many countries have promoted waste incineration for power generation. Currently, the household waste incineration rate has reached 70%-80% in Japan, Denmark, and Switzerland [2]. However, due to technical, technological and geographical location, if waste incineration of toxic waste gas power generation is not effectively treated, it will seriously threaten the lives and health of residents. Therefore, people still have doubts about the waste treatment method of the incineration for power generation, which hinders the construction of waste incineration plants.

Landfill technology is mature, simple process and low processing capacity and costs, and to better achieve harmless the surface. It is currently an important method of centralized disposal of urban waste [5]. In addition, the wastes obtained through other methods of disposal of garbage, such as ash from incineration, excess compost, and non-recyclable materials, still will be returned to the environment in the form of landfills [6]. Landfill is the main method of waste disposal, but its large area. And there are secondary pollutions such as landfill leachate polluting groundwater and soil, and odor generated by garbage dumping affects the air quality around the site. In some developing countries, due to unreasonable urban planning, there are still a large number of residents living around the landfill, which directly affects the living environment of nearby residents [7]. In addition, the site selection of landfill is quite complicated, which needs to consider the constraints of local economy, transportation, geographical and topographical conditions, climate, environmental geological conditions, surface hydrological conditions, hydrogeological engineering conditions, etc. In addition, the site selection of waste landfills is quite complicated, and needs to consider the constraints of local economy, transportation, climate, geographical and topographical conditions, environmental geological conditions, and surface hydrological conditions [8]. Site selection is very important in the construction of waste landfills. In order to minimize social, environmental and economic costs, many scholars have conducted research on the site selection and environmental impact assessment of waste disposal sites. Jiang et al. [9] established a site selection model for municipal solid waste incineration plants by examining evaluation factors such as economy, environmental protection, population size, and operating costs, combined with a swarm optimization algorithm. Zarin et al. [10] combined geographic information system with fuzzy logic, analytic hierarchy process and weighted linear combination method based on multi-criteria decision-making, and provided a new method for solid waste landfill site selection in complex terrain areas. Rezaeisabzevar et al. [11] discussed various methods of landfill site selection, including ordered weighted averaging, analytic network process, TODIM and grey systems theory. Eghtesadifard et al. [12] used K-Means and multi-attribute decision analysis to establish a comprehensive algorithm for the selection of municipal solid waste landfills.

Scientific and rational site selection methods can reduce the impact of landfills on the environment and society. However, due to the many factors affecting the site selection of waste landfills and the limited cognitive ability of decision makers, there is a large amount of uncertain information in the site selection process. In order to describe the uncertain information in practical problems, Zadeh proposed fuzzy set theory [13]. With the development and improvement of fuzzy set theory, some scholars have conducted research on the extended forms of fuzzy sets, such as intuitionistic fuzzy sets [14], interval fuzzy sets [15], hesitant fuzzy sets [16] and so on. The fuzzy set (FS) theory proposed by Zadeh only describes the fuzziness of information from the perspective of the degree of membership. The intuitionistic fuzzy set (IFS) proposed by Atanassov [14] describes the fuzziness of information more completely from the perspective of membership and non-membership. In order to describe a wider range of fuzzy information, Yager [17] proposed the Pythagorean fuzzy set (PFS), which can describe the situation where the sum of the degree of membership and the degree of non-membership exceeds 1, and the sum of the squares does not exceed 1. However, there are some situations in practice that PFS can’t describe. Yager further promoted PFS and proposed the concept of q-rung orthopair fuzzy set (q-ROFS) [18]. q-ROFS is a generalized form of IFS and PFS, which can describe a wider range of uncertain phenomena. In the actual decision-making process, there is often hesitation. For this reason, Torra [16] proposed the hesitant fuzzy set (HFS), which allows the membership of an element to be a set of multiple possible values between 0 and 1. HFS can more comprehensively describe the uncertain information given by the decision maker, but the elements in its set cannot be repeated, and there is no difference between them. However, in most cases, due to the personal preference of the decision makers and the number of decision makers, different degrees of membership may have different importance. The hesitant fuzzy element cannot describe the preference information of decision makers for different degrees of membership. This problem also exists in q-rung orthopair hesitant fuzzy sets (q-ROHFS) [19]. Bedregal et al. [20] tried to use fuzzy multi-set to solve this problem, but its expression was too cumbersome. In order to overcome the shortcomings of HFS and at the same time solve the cumbersome problem of fuzzy multi-set representation, Xu et al. [21] first proposed the probabilistic hesitant fuzzy set (PHFS). But it requires that the sum of the probabilities of the probabilistic hesitant fuzzy elements is equal to 1, which leads to the limitation of the expression space of the decision maker. Zhang et al. [22] improved the probabilistic hesitant fuzzy set, weakened its constraint conditions, and allowed the sum of the probabilities of probabilistic hesitant fuzzy elements to be less than 1.

There are many factors that affect the site selection of waste landfills. Therefore, decision makers will inevitably give too high or too low evaluation values due to lack of personal experience or prejudice towards things. Yager [23] proposed the power average (PA) operator, which reduces the negative impact of unreasonable evaluation information on the results by considering the support relationship between the data. Considering the powerful functions of the PA operator, some scholars have done further research on the PA operator and widely used it in intuitionistic fuzzy information integration [24], hesitant fuzzy information integration [25], and language information integration [26]. In the evaluation process of landfill site selection, there is an association between different influencing factors, so that the determination of the evaluation value of one factor will be affected by other factors. If this is not considered, it will affect the final decision result. For multi-attribute decision in evaluating the relationship between the information, the majority of scholars a lot of work. Choquet integral operator, Bonferroni mean (BM) operator, Heronian mean (HM) operator, Maclaurin symmetric mean (MSM) operator, and Muirhead mean (MM) operator are successively used for information integration [27–31]. Among them, the MM operator can reflect the correlation relationship between any number of decision information. In order to solve the negative impact of unreasonable evaluation information on the results, and to characterize the internal relationship between different factors, He et al. [32] tried to combine the PA operator with the BM operator and proposed the PBM operator. Subsequently, scholars successively proposed PHM operators, PMSM, and PMM operators, and used them to solve multi-attribute decision making problems in various environments [33–35]. In addition, Iampan et al. [36] proposed a multi-attribute decision making method based on LDFEWA operator, which relaxed the strict constraints of IFS, PFS and q-ROFS by considering reference/control parameters. Riaz et al. [37] proposed a new multicriteria decision-making approach based on BPFWG operator to address uncertain real-life situations. Ramakrishnan et al. [38] integrated the Cloud model with the technique for order of preference by similarity to an ideal solution to solve the multi-attribute decision making problem.

In summary, the research on site selection of waste landfill based on fuzzy theory has made certain progress. However, the current presentation of evaluation information based on FS and IFS is not complete, and the hesitation of decision makers is not considered. In order to describe the evaluation information of decision makers more completely, this paper is inspired by the probabilistic hesitant fuzzy set [22], and improves the q-rung orthopair hesitant fuzzy sets [19], and proposes the q-rung orthopair probabilistic hesitant fuzzy set (q-ROPHFS). q-ROPHFS not only describe the evaluation information of decision-makers more completely, but also give decision-makers more freedom to make decisions. Compared with IFS, q-ROPHFS has a wider range of membership and non-membership. In addition, in the evaluation process of landfill site selection, there are many factors that affect the evaluation of candidate sites, and the evaluation information given by decision makers is not completely accurate. At the same time, the evaluation value of each influencing factor will be affected by other factors. At present, most studies have not considered these issues. In order to eliminate the adverse effects of unreasonable information given by decision makers in the evaluation process, and to better characterize the correlation between evaluation information, PA operator and MM operator are extended to q-ROPHFS. Then, the q-rung orthopair probabilistic hesitant fuzzy power weight Muirhead mean (q-ROPHFPWMM) operator is constructed and applied to the multi-attribute decision-making algorithm for landfill site selection.

The proposed algorithm has great advantages. Compared with other methods based on FS and IFS [39,40], the proposed method considers decision-maker’s hesitation and creatively uses probability to represent it. In addition, the Muirhead average operator is extended to q-rung orthopair probabilistic hesitation fuzzy set, and the q-ROPHFPWMM operator is proposed. Because operators can degenerate into other operators by taking different parameter vectors, operators have the advantage of multiple operators. Meanwhile, a q-ROPHFPWMM operator based multi-attribute decision making algorithm is proposed. Compared with other methods, this algorithm has good robustness.

## 2. Preliminaries

### 2.1 The definition of q-ROHFS and PHFS

**Definition 1.** [19] Let *X* = {*x*_1_,*x*_2_,⋯,*x*_*n*_} be a fixed set, then the q-rung orthopair hesitant fuzzy set (q-ROHFS) defined on *X* can be represented as:

$$A=\{<x,\Gamma_{A},\Psi_{A}{>}_{q}|x\in X\},$$

where Γ_*A*_ and Ψ_*A*_ respectively represent the set of all the membership degrees and non-membership degrees of the element *x*(*x*∈*X*) belonging to the set *A*, and for ∀*x*∈*X*, ∀*μ*∈Γ_*A*_, ∀*υ*∈Ψ_*A*_, satisfy 0<*μ*,*υ*<1, *μ*^*q*^+*υ*^*q*^≤1.

**Definition 2.** [21] Let *X* = {*x*_1_,*x*_2_,⋯,*x*_*n*_} be a fixed set, then the probabilistic hesitant fuzzy set (PHFS) defined on *X* can be represented as:

A={<x,μ(p)>|x∈X},

where *μ* represents the possible membership degree of the element *x*(*x*∈*X*) belonging to the set *A*, *p* is the probability corresponding to it, and for ∀*x*∈*X*, satisfy 0<*μ*<1, 0<*p*<1.

### 2.2 PA operator and MM operator

**Definition 3.** [22] -Let *a*_*i*_(*i* = 1,2,⋯,*n*) be a set of non-negative real numbers, then the power average (PA) operator is defined as:

$$PA(a_{1},a_{2},\cdots ,a_{n})=\frac{\sum_{i=1}^{n}(1+T(a_{i}))a_{i}}{\sum_{j=1}^{n}\left(1+T(a_{j})\right)},$$

wherein, $T(a_{i})=\sum_{j=1,j\neq i}^{n}Sup(a_{i},a_{j})$, *Sup*(*a*_*i*_,*a*_*j*_) represent the degree of support between *a*_*i*_ and *a*_*j*_, and satisfy the following conditions:

1. *Sup*(*a*_*i*_,*a*_*j*_)∈[0,1];
2. *Sup*(*a*_*i*_,*a*_*j*_) = *Sup*(*a*_*j*_,*a*_*i*_);
3. *Sup*(*a*,*b*)≥*Sup*(*c*,*d*), if and only if |*a*−*b*|≥|*c*−*d*|.

**Definition 4.** [41] Let *a*_*i*_(*i* = 1,2,⋯,*n*) be a set of non-negative real numbers and *P* = (*p*_1_,*p*_2_,⋯,*p*_*n*_)∈*R*^*n*^ is a parameter vector, then the Muirhead Mean (MM) operator is defined as:

$$MM^{P}(a_{1},a_{2},\cdots ,a_{n})={\left(\frac{1}{n!}\sum_{\vartheta \in S_{n}}\prod_{j=1}^{n}a_{\vartheta (j)}^{p_{{}_{j}}}\right)}^{\frac{1}{\sum_{j=1}^{n}p_{{}_{j}}}},$$

where *ϑ* = {*ϑ*(1),*ϑ*(2),⋯*ϑ*(*n*)} is arbitrary sequence of (1,2,⋯,*n*), and *S*_*n*_ is the set of all possible sequences of (1,2,⋯,*n*).

## 3. The q-rung orthopair probabilistic hesitant fuzzy set

### 3.1 The definition of q-ROPHFS

**Definition 5.** Let *X* = {*x*_1_,*x*_2_,⋯,*x*_*n*_} be a fixed set, then the q-rung orthopair probabilistic hesitant fuzzy set (q-ROPHFS) defined on *X* can be represented as:

$$A=\{<x,\Gamma_{A},\Psi_{A}>|x\in X\},$$

where Γ_*A*_ = {*μ*_1_(*p*_1_),*μ*_2_(*p*_2_),⋯,*μ*_1_(*p*_1_)}, $\Psi_{A}=\{\upsilon_{1}({\bar{p}}_{1}),\upsilon_{2}({\bar{p}}_{2}),\cdots ,\upsilon_{m}({\bar{p}}_{m})\}$. *μ*_*l*_ and *υ*_*m*_ respectively represent the possible membership degree and possible non-membership degree of element *x*(*x*∈*X*) belonging to the set *A*. *p*_*l*_ and ${\bar{p}}_{m}$ are the corresponding probabilities.

In addition, for ∀*x*∈*X*, ∀*μ*∈Γ_*A*_, ∀*υ*∈Ψ_*A*_, satisfies the following conditions,

$$0<\mu <1,\;0<\upsilon <1,\;\mu^{q}+\upsilon^{q}\leq 1,$$

$$0<p_{l}<1,\;0<{\bar{p}}_{m}<1,\;\sum_{l=1}^{\left|\Gamma_{A}\right|}p_{l}\leq 1,\;\sum_{m=1}^{\left|\Psi_{A}\right|}{\bar{p}}_{m}\leq 1,$$

Where |Γ_*A*_| and |Ψ_*A*_| represent the number of elements contained in them, respectively.

For convenience, *h* = <Γ_*h*_,Ψ_*h*_> is called q-rung orthopair probabilistic hesitatnt fuzzy element (q-ROPHFE).

### 3.2 The operation of q-ROPHFE

The operation of q-ROPHFE is defined by referring to the operation of q-rung orthopair hesitant fuzzy element [19] and the operation of probabilistic hesitant fuzzy element [21].

**Definition 6.** Let *h* = <Γ_*h*_,Ψ_*h*_>, $h_{1}=<\Gamma_{h_{1}},\Psi_{h_{1}}>$ and $h_{2}=<\Gamma_{h_{2}},\Psi_{h_{2}}>$ be three q-ROPHFEs, and *λ* be arbitrary positive number, then the definition operation is as follows:

$$h_{1}⊕h_{2}=\langle \underset{\begin{array}{l} \mu_{1_{l}}\in \Gamma_{h_{1}}\\ \mu_{2_{k}}\in \Gamma_{h_{2}} \end{array}}{\cup }\{\left[{\left(\mu_{1_{l}}^{q}+\mu_{2_{k}}^{q}-\mu_{1_{l}}^{q}\mu_{2_{k}}^{q}\right)}^{1/q}\right](\frac{p_{1_{l}}p_{2_{k}}}{\sum_{l=1}^{\left|\Gamma_{h_{1}}\right|}p_{1_{l}}\cdot \sum_{k=1}^{\left|\Gamma_{h_{2}}\right|}p_{2_{k}}})\},\underset{\begin{array}{l} v_{1_{m}}\in \Psi_{h_{1}}\\ v_{2_{n}}\in \Psi_{h_{2}} \end{array}}{\cup }\{\left[\upsilon_{1_{m}}\upsilon_{2_{n}}\right](\frac{{\bar{p}}_{1_{m}}{\bar{p}}_{2_{n}}}{\sum_{m=1}^{\left|\Psi_{h_{1}}\right|}{\bar{p}}_{1_{m}}\cdot \sum_{n=1}^{\left|\Psi_{h_{2}}\right|}{\bar{p}}_{2_{n}}})\}\rangle$$ (1)

$$h_{1}⊗h_{2}=\langle \underset{\begin{array}{l} \mu_{1_{l}}\in \Gamma_{h_{1}}\\ \mu_{2_{k}}\in \Gamma_{h_{2}} \end{array}}{\cup }\{\left[\mu_{1_{l}}\mu_{2_{k}}\right](\frac{p_{1_{l}}p_{2_{k}}}{\sum_{l=1}^{\left|\Gamma_{h_{1}}\right|}p_{1_{l}}\cdot \sum_{k=1}^{\left|\Gamma_{h_{2}}\right|}p_{2_{k}}})\},\underset{\begin{array}{l} v_{1_{m}}\in \Psi_{h_{1}}\\ v_{2_{n}}\in \Psi_{h_{2}} \end{array}}{\cup }\{\left[{\left(v_{1_{m}}^{q}+v_{2_{n}}^{q}-v_{1_{m}}^{q}v_{2_{n}}^{q}\right)}^{1/q}\right](\frac{{\bar{p}}_{1_{m}}{\bar{p}}_{2_{n}}}{\sum_{m=1}^{\left|\Psi_{h_{1}}\right|}{\bar{p}}_{1_{m}}\cdot \sum_{n=1}^{\left|\Psi_{h_{2}}\right|}{\bar{p}}_{2_{n}}})\}\rangle$$ (2)

$$\lambda h=\langle \underset{\mu_{l}\in \Gamma_{h}}{\cup }\{\left[{\left(1-{\left(1-\mu_{l}^{q}\right)}^{\lambda }\right)}^{1/q}](p_{l}\right)\},\underset{v_{m}\in \Psi_{h}}{\cup }\{v_{m}^{\lambda }({\bar{p}}_{m})\}\rangle$$ (3)

$$h^{\lambda }=\langle \underset{\mu_{l}\in \Gamma_{h}}{\cup }\{\mu_{l}^{\lambda }(p_{l})\},\underset{v_{m}\in \Psi_{h}}{\cup }\{\left[{\left(1-{\left(1-v_{m}^{q}\right)}^{\lambda }\right)}^{1/q}]({\bar{p}}_{m}\right)\}\rangle$$ (4)

$$h^{c}=<\Psi_{h},\Gamma_{h}>$$ (5)

### 3.3 The distance between two q-ROPHFEs

Distance measure is a commonly used tool to describe the difference between the two. In this section, we propose the distance between any two q-ROPHFEs

**Definition 7.** Let *h*_1_ and *h*_2_ be two q-ROPHFEs. If *d*(*h*_1_,*h*_2_) is the distance between *h*_1_ and *h*_2_, it needs to meet the following conditions:

1. 0≤*d*(*h*_1_,*h*_2_)≤1;
2. If and only if *h*_1_ = *h*_2_, *d*(*h*_1_,*h*_2_) = 0;
3. *d*(*h*_1_,*h*_2_) = *d*(*h*_2_,*h*_1_).

Based on the Hamming distance and Euclidean distance, the Hamming distance and Euclidean distance between two q-ROPHFEs are defined as follows.

1. The standardized Hamming distance between *h*_1_ and *h*_2_ is:
$$d_{H}(h_{1},h_{2})=\frac{1}{2}(\sum_{i=1}^{l_{\Gamma }}\left|p^{*}({\left(\mu_{1}^{\theta (i)}\right)}^{q}-{\left(\mu_{2}^{\theta (i)}\right)}^{q})\right|+\sum_{j=1}^{l_{\Psi }}\left|{\bar{p}}^{*}({\left(\upsilon_{1}^{\theta (j)}\right)}^{q}-{\left(\upsilon_{2}^{\theta (j)}\right)}^{q})\right|)$$
  wherein, p* is the maximum value that makes $\frac{p_{1}^{\sigma (i)}}{p^{*}}$ and $\frac{p_{2}^{\sigma (i)}}{p^{*}}$ a positive integer, and ${\bar{p}}^{*}$ is the maximum value that makes $\frac{{\bar{p}}_{1}^{\sigma (i)}}{{\bar{p}}^{*}}$ and $\frac{{\bar{p}}_{2}^{\sigma (i)}}{{\bar{p}}^{*}}$ a positive integer. For $\forall \mu_{1}^{\sigma (i)}(p_{1}^{\sigma (i)})\in \Gamma_{h_{1}}$, if $p_{1}^{\sigma (1)}/p^{*}=n$, then $\mu_{1}^{\theta (1)}=\cdots =\mu_{1}^{\theta (n)}=\mu_{1}^{\sigma (1)}$; If $p_{1}^{\sigma (2)}/p^{*}=m$, then $\mu_{1}^{\theta (n+1)}=\cdots =\mu_{1}^{\theta (n+m)}=\mu_{1}^{\sigma (2)}$. And so on, you get $\mu_{1}^{\theta (i)}(i=1,2,\cdots ,l_{\Gamma })$. $\mu_{1}^{\sigma (i)}$ is the ith largest value in $\Gamma_{h_{1}}$, which satisfies $\mu_{1}^{\sigma (1)}>\mu_{1}^{\sigma (2)}>\cdots >\mu_{1}^{\sigma (i)}>\cdots >\mu_{1}^{\sigma (|\Gamma_{h_{1}}|)}$. And similarly, you get $\mu_{2}^{\theta (i)}(i=1,2,\cdots ,l_{\Gamma })$, $\upsilon_{1}^{\theta (j)}(j=1,2,\cdots ,l_{\Psi })$ and $\upsilon_{2}^{\theta (j)}(j=1,2,\cdots ,l_{\Psi })$.
2. The standardized Euclidean distance between *h*_1_ and *h*_2_ is:
$$d_{E}(h_{1},h_{2})=\sqrt{\frac{1}{2}(\sum_{i=1}^{l_{\Gamma }}{\left|p^{*}({\left(\mu_{1}^{\theta (i)}\right)}^{q}-{\left(\mu_{2}^{\theta (i)}\right)}^{q})\right|}^{2}+\sum_{j=1}^{l_{\Psi }}{\left|{\bar{p}}^{*}({\left(\upsilon_{1}^{\theta (j)}\right)}^{q}-{\left(\upsilon_{2}^{\theta (j)}\right)}^{q})\right|}^{2})}$$
3. The standardized generalized distance between *h*_1_ and *h*_2_ is:
$$d_{G}(h_{1},h_{2})={\left(\frac{1}{2}(\sum_{i=1}^{l_{\Gamma }}{\left|p^{*}({\left(\mu_{1}^{\theta (i)}\right)}^{q}-{\left(\mu_{2}^{\theta (i)}\right)}^{q})\right|}^{\lambda }+\sum_{j=1}^{l_{\Psi }}{\left|{\bar{p}}^{*}({\left(\upsilon_{1}^{\theta (j)}\right)}^{q}-{\left(\upsilon_{2}^{\theta (j)}\right)}^{q})\right|}^{\lambda })\right)}^{\frac{1}{\lambda }}$$

In particular, when *λ* = 1, the generalized distance of q-ROPHFE degenerates to the Hamming distance of q-ROPHFE, and when *λ* = 2, it degenerates to the Euclidean distance of q-ROPHFE.

In most cases, $\sum_{i=1}^{\left|\Gamma_{h_{1}}\right|}p_{1}^{\sigma (i)}\neq \sum_{i=1}^{\left|\Gamma_{h_{2}}\right|}p_{2}^{\sigma (i)}$. In order to facilitate the calculation, it is necessary to add elements to the set with a small sum of probability. For example, if $\sum_{i=1}^{\left|\Gamma_{h_{1}}\right|}p_{1}^{\sigma (i)}>\sum_{i=1}^{\left|\Gamma_{h_{2}}\right|}p_{2}^{\sigma (i)}$, then add element $\bar{\alpha }(p^{*})=[\zeta \alpha^{+}+(1-\zeta )\alpha^{-}](p^{*})$ to $\Gamma_{h_{2}}$. Wherein, $\alpha^{+}=\underset{\mu (p)\in \Gamma_{h_{2}}}{\max}\{\mu \}$, $\alpha^{-}=\underset{\mu (p)\in \Gamma_{h_{2}}}{\min}\{\mu \}$, *ζ* is the risk appetite coefficient. For example, (1) If the decision-maker is risk-neutral, then *ζ* = 0.5, $\bar{\alpha }=0.5\alpha^{+}+0.5\alpha^{-}$; (2) If the decision-maker is risk-averse, then *ζ* = 0, $\bar{\alpha }=\alpha^{-}$; (3) If the decision-maker is a risk-preferred, then *ζ* = 1, $\bar{\alpha }=\alpha^{+}$.

We do the same thing for $\Psi_{h_{1}}$ and $\Psi_{h_{2}}$.

**Example 1.** There are two q-ROPHFEs *h*_1_ =〈{0.5|0.6,0.4|0.3},{0.7|0.5,0.6|0.5}〉 and *h*_2_ =〈{0.6|0.5,0.5|0.5},{0.5|0.4,0.4|0.4}〉. Obviously, $\sum_{i=1}^{\left|\Gamma_{h_{1}}\right|}p_{1}^{\sigma (i)}<\sum_{i=1}^{\left|\Gamma_{h_{2}}\right|}p_{2}^{\sigma (i)}$. Then, we need to add elements to $\Gamma_{h_{1}}$. Assuming that the decision maker is risk-averse, then *ζ* = 0. After adding the element, $\Gamma_{h_{1}}=\{0.5|0.6,0.4|0.3,0.4|0.1\}$. Similarly, $\Psi_{h_{2}}=\{0.5|0.4,0.4|0.4,0.4|0.2\}$ can be obtained.

### 3.4 The ranking of the q-ROPHFEs

Inspired by probabilistic hesitant fuzzy set [21], we define the score function and deviation degree of q-ROPHFE.

**Definition 8.** Let *h* be a q-ROPHFE, then its score function *S*(*h*) is defined below:

$$S(h)=\frac{1}{2}(\frac{\sum_{l=1}^{\left|\Gamma_{h}\right|}\mu_{l}^{q}\cdot p_{l}}{\sum_{l=1}^{\left|\Gamma_{h}\right|}p_{l}}-\frac{\sum_{m=1}^{\left|\Psi_{h}\right|}(1-\upsilon_{m}^{q})\cdot {\bar{p}}_{m}}{\sum_{m=1}^{\left|\Psi_{h}\right|}{\bar{p}}_{m}}),$$

where |Γ_*h*_| and |Ψ_*h*_| represent the number of elements contained in them respectively.

Suppose that *h*_1_ and *h*_2_ are two arbitrary q-ROPHFEs. If *S*(*h*_1_)>*S*(*h*_2_), then *h*_1_ is considered to be superior to *h*_2_, denoted as *h*_1_≻*h*_2_. If *S*(*h*_1_) = *S*(*h*_2_), there is no way to compare *h*_1_ and *h*_2_ using a score function. Therefore, the deviation degree of q-ROPHFE needs to be defined.

**Definition 9.** Let *h* be a q-ROPHFE, and its score function is expressed by *η*, then the deviation degree *D*(*h*) is defined below:

$$D(h)=\frac{1}{2}(\frac{\sum_{l=1}^{\left|\Gamma_{h}\right|}p_{l}{\left(\mu_{l}^{q}-\eta^{q}\right)}^{2}}{\sum_{l=1}^{\left|\Gamma_{h}\right|}p_{l}}+\frac{\sum_{m=1}^{\left|\Psi_{h}\right|}{\bar{p}}_{m}{\left((1-\upsilon_{m}^{q})-\eta^{q}\right)}^{2}}{\sum_{m=1}^{\left|\Psi_{h}\right|}{\bar{p}}_{m}}).$$

The comparison method of q-ROPHFE is given according to the score function and deviation degree of q-ROPHFE:

(1) If *S*(*h*_1_)>*S*(*h*_2_), then *h*_1_≻*h*_2_;
(2) If *S*(*h*_1_) = *S*(*h*_2_),
  (2.1) If *D*(*h*_1_)>*D*(*h*_2_), then *h*_1_≺*h*_2_;
  (2.2) If *D*(*h*_1_) = *D*(*h*_2_), then *h*_1_ = *h*_2_.

**Example 2.** There are two q-ROPHFEs *h*_1_ =〈{0.5|0.6,0.4|0.3},{0.7|0.5,0.6|0.5}〉 and *h*_2_ =〈{0.6|0.5,0.5|0.5},{0.5|0.4,0.4|0.4}〉.Calculate their score function, we can get *S*(*h*_1_) = 0.4083, *S*(*h*_2_) = 0.5500. Therefore, *h*_1_≺*h*_2_.

## 4. The q-rung orthopair probabilistic hesitant fuzzy power Muirhead mean operator

### 4.1 The definition of the q-ROPHFPWMM operator

In this section, we generalize the power average operator [22] and Muirhead mean operator [41] to q-ROPHFS, and propose the q-rung orthopair probabilistic hesitant fuzzy power Muirhead mean operator.

**Definition 10.** Let *h*_*i*_(*i* = 1,2,⋯,*n*) be a set of q-ROPHFEs and *R* = (*r*_1_,*r*_2_,⋯,*r*_*n*_)∈*R*^*n*^ be a vector of parameters, and ***ω*** = (*ω*_1_,*ω*_2_,⋯,*ω*_*n*_) be weight vectors that satisfy *ω*_*i*_ = [0,1] and $\sum_{i=1}^{n}\omega_{1}=1$, then the q-rung probabilistic hesitant fuzzy power weight Muirhead mean (q-ROPHFPMM) operator is defined as follows:

$$q-PHFPMM^{R}(h_{1},h_{2},\cdots ,h_{n})={\left(\frac{1}{n!}\underset{\vartheta \in S_{n}}{⊕}\overset{n}{\underset{i=1}{⊗}}{\left(n\frac{\omega_{\vartheta (i)}(1+T(h_{\vartheta (i)}))}{\sum_{j=1}^{n}\omega_{j}(1+T(h_{j}))}h_{\vartheta (i)}\right)}^{r_{i}}\right)}^{\frac{1}{\sum_{i=1}^{n}r_{i}}}$$

Wherein, $T(h_{i})=\sum_{j=1,j\neq i}^{n}Sup(h_{i},h_{j})$, *Sup*(*h*_*i*_,*h*_*j*_) = 1−*d*(*h*_*i*_,*h*_*j*_). *ϑ* = {*ϑ*(1),*ϑ*(2),⋯*ϑ*(*n*)} represents any permutation of (1,2,⋯,*n*), *S*_*n*_ represents the set of all possible permutations of (1,2,⋯,*n*), and *n* is the regulation coefficient.

To simplify the equation, let $\varpi_{i}=\frac{\omega_{i}(1+T(h_{i}))}{\sum_{j=1}^{n}\omega_{j}(1+T(h_{j}))}$, then Eq (6) is simplified as follows:

$$q-PHFPMM^{R}(h_{1},h_{2},\cdots ,h_{n})={\left(\frac{1}{n!}\underset{\vartheta \in S_{n}}{⊕}\overset{n}{\underset{i=1}{⊗}}{\left(n\varpi_{\vartheta (i)}h_{\vartheta (i)}\right)}^{r_{i}}\right)}^{\frac{1}{\sum_{j=1}^{n}r_{i}}}$$

**Theorem 1.** Let *h*_*i*_(*i* = 1,2,⋯,*n*) be a set of q-ROPHFEs, *R* = (*r*_1_,*r*_2_,⋯,*r*_*n*_)∈*R*^*n*^ be a vector of parameters and ***ω*** = (*ω*_1_,*ω*_2_,⋯,*ω*_*n*_) be weight vectors that satisfy *ω*_*i*_ = [0,1] and $\sum_{i=1}^{n}\omega_{1}=1$. The aggregated value using q-PHFPMM operator is still q-ROPHFE, and

$$\begin{array}{c} q-PHFPMM^{R}(h_{1},h_{2},\cdots ,h_{n})=\\ \langle \underset{\begin{array}{c} \mu_{\vartheta {\left(i\right)}_{l}}\in \Gamma_{h_{\vartheta (i)}}\\ i=1,2,\cdots n \end{array}}{\cup }\{\left[{\left({\left(1-\prod_{\vartheta \in S_{n}}{\left(1-\prod_{i=1}^{n}{\left(1-{\left(1-\mu_{\vartheta (i)}^{q}\right)}^{n\varpi_{\vartheta (i)}}\right)}^{r_{i}}\right)}^{1/n!}\right)}^{1/q}\right)}^{1/\sum_{i=1}^{n}r_{i}}](\frac{\prod_{\vartheta \in S_{n}}\prod_{i=1}^{n}p_{\vartheta {\left(i\right)}_{l}}}{\prod_{\vartheta \in S_{n}}\prod_{i=1}^{n}\sum_{l=1}^{\left|\Gamma_{h_{\vartheta (i)}}\right)}p_{\vartheta {\left(i\right)}_{l}}}\right)\},\\ \underset{\begin{array}{c} v_{\vartheta {\left(i\right)}_{m}}\in \Psi_{h_{\vartheta (i)}}\\ i=1,2,\cdots n \end{array}}{\cup }\{\left[{\left(1-{\left(1-\prod_{\vartheta \in S_{n}}{\left(1-\prod_{i=1}^{n}{\left(1-\upsilon_{\vartheta (i)}^{qn\varpi_{\vartheta (i)}}\right)}^{r_{i}}\right)}^{1/n!}\right)}^{1/\sum_{i=1}^{n}r_{i}}\right)}^{1/q}](\frac{\prod_{\vartheta \in S_{n}}\prod_{i=1}^{n}{\bar{p}}_{\vartheta {\left(i\right)}_{m}}}{\prod_{\vartheta \in S_{n}}\prod_{i=1}^{n}\sum_{m=1}^{\left|\Psi_{h_{\vartheta (i)}}\right)}{\bar{p}}_{\vartheta {\left(i\right)}_{m}}}\right)\}\rangle \end{array}$$

**Proof.**

According to the operation of q-ROPHFEs in Definition 6, we can get

$$n\varpi_{\vartheta (i)}h_{\vartheta (i)}=\langle \underset{\mu_{\vartheta {\left(i\right)}_{l}}\in \Gamma_{h_{\vartheta (i)}}}{\cup }\{\left[{\left(1-{\left(1-\mu_{\vartheta {\left(i\right)}_{l}}^{q}\right)}^{n\varpi_{\vartheta (i)}}\right)}^{1/q}](p_{\vartheta {\left(i\right)}_{l}}\right)\},\underset{v_{\vartheta {\left(i\right)}_{m}}\in \Psi_{h_{\vartheta (i)}}}{\cup }\{\left[\upsilon_{\vartheta {\left(i\right)}_{m}}^{n\varpi_{\vartheta (i)}}]({\bar{p}}_{\vartheta {\left(i\right)}_{m}}\right)\}\rangle$$

and

$${\left(n\varpi_{\vartheta (i)}h_{\vartheta (i)}\right)}^{r_{i}}=\langle \underset{\mu_{\vartheta {\left(i\right)}_{l}}\in \Gamma_{h_{\vartheta (i)}}}{\cup }\{\left[{\left({\left(1-{\left(1-\mu_{\vartheta {\left(i\right)}_{l}}^{q}\right)}^{n\varpi_{\vartheta (i)}}\right)}^{1/q}\right)}^{r_{i}}](p_{\vartheta {\left(i\right)}_{l}}\right)\},\underset{\upsilon_{\vartheta {\left(i\right)}_{m}}\in \Psi_{h_{\vartheta (i)}}}{\cup }\{\left[{\left(1-{\left(1-\upsilon_{\vartheta {\left(i\right)}_{m}}^{n\varpi_{\vartheta (i)}}\right)}^{r_{i}}\right)}^{1/q}]({\bar{p}}_{\vartheta {\left(i\right)}_{m}}\right)\}\rangle$$

Then, we use mathematical induction theory to get

$$\begin{array}{c} \overset{n}{\underset{i=1}{⊗}}{\left(n\varpi_{\vartheta (i)}h_{\vartheta (i)}\right)}^{r_{i}}=\langle \underset{\begin{array}{c} \mu_{\vartheta {\left(i\right)}_{l}}\in \Gamma_{h_{\vartheta (i)}}\\ i=1,2,\cdots n \end{array}}{\cup }\{\left[\prod_{i=1}^{n}{\left({\left(1-{\left(1-\mu_{\vartheta {\left(i\right)}_{l}}^{q}\right)}^{n\varpi_{\vartheta (i)}}\right)}^{1/q}\right)}^{r_{i}}](\frac{\prod_{i=1}^{n}p_{\vartheta {\left(i\right)}_{l}}}{\prod_{i=1}^{n}\sum_{l=1}^{\left|\Gamma_{h_{\vartheta (i)}}\right|}p_{\vartheta {\left(i\right)}_{l}}}\right)\},\\ \underset{\begin{array}{c} v_{\vartheta {\left(i\right)}_{m}}\in \Psi_{h_{\vartheta (i)}}\\ i=1,2,\cdots n \end{array}}{\cup }\{\left[{\left(1-\prod_{i=1}^{n}{\left(1-\upsilon_{\vartheta {\left(i\right)}_{m}}^{n\varpi_{\vartheta (i)}}\right)}^{r_{i}}\right)}^{1/q}](\frac{\prod_{i=1}^{n}{\bar{p}}_{\vartheta {\left(i\right)}_{m}}}{\prod_{i=1}^{n}\sum_{m=1}^{\left|\Psi_{h_{\vartheta (i)}}\right|}{\bar{p}}_{\vartheta {\left(i\right)}_{m}}}\right)\}\rangle \end{array}$$

Because of $\sum_{\vartheta \in S_{n}}\frac{\prod_{i=1}^{n}p_{\vartheta {\left(i\right)}_{l}}}{\prod_{i=1}^{n}\sum_{l=1}^{\left|\Gamma_{h_{\vartheta (i)}}\right|}p_{\vartheta {\left(i\right)}_{l}}}=1$, $\sum_{\vartheta \in S_{n}}\frac{\prod_{i=1}^{n}{\bar{p}}_{\vartheta {\left(i\right)}_{m}}}{\prod_{i=1}^{n}\sum_{m=1}^{\left|\Psi_{h_{\vartheta (i)}}\right|}{\bar{p}}_{\vartheta {\left(i\right)}_{m}}}=1$, we can further use mathematical induction to get

$$\begin{array}{c} \underset{\vartheta \in S_{n}}{⊕}\overset{n}{\underset{i=1}{⊗}}{\left(n\varpi_{\vartheta (i)}h_{\vartheta (i)}\right)}^{r_{i}}=\langle \underset{\begin{array}{c} \mu_{\vartheta {\left(i\right)}_{l}}\in \Gamma_{h_{\vartheta (i)}}\\ i=1,2,\cdots n \end{array}}{\cup }\{\left[{\left(1-\prod_{\vartheta \in S_{n}}\left(1-\prod_{i=1}^{n}{\left(1-{\left(1-\mu_{\vartheta {\left(i\right)}_{l}}^{q}\right)}^{n\varpi_{\vartheta (i)}}\right)}^{r_{i}}\right)\right)}^{1/q}](\frac{\prod_{\vartheta \in S_{n}}\prod_{i=1}^{n}p_{\vartheta {\left(i\right)}_{l}}}{\prod_{\vartheta \in S_{n}}\prod_{i=1}^{n}\sum_{l=1}^{\left|\Gamma_{h_{\vartheta (i)}}\right|}p_{\vartheta {\left(i\right)}_{l}}}\right)\},\\ \underset{\begin{array}{c} v_{\vartheta {\left(i\right)}_{m}}\in \Psi_{h_{\vartheta (i)}}\\ i=1,2,\cdots n \end{array}}{\cup }\{\left[\prod_{\vartheta \in S_{n}}{\left(1-\prod_{i=1}^{n}{\left(1-\upsilon_{\vartheta {\left(i\right)}_{m}}^{n\varpi_{\vartheta (i)}}\right)}^{r_{i}}\right)}^{1/q}](\frac{\prod_{\vartheta \in S_{n}}\prod_{i=1}^{n}{\bar{p}}_{\vartheta {\left(i\right)}_{m}}}{\prod_{\vartheta \in S_{n}}\prod_{i=1}^{n}\sum_{m=1}^{\left|\Psi_{h_{\vartheta (i)}}\right|}{\bar{p}}_{\vartheta {\left(i\right)}_{m}}}\right)\}\rangle \end{array}.$$

Similarly, according to Definition 6, we can get

$$\begin{array}{c} \frac{1}{n!}\underset{\vartheta \in S_{n}}{⊕}\overset{n}{\underset{i=1}{⊗}}{\left(n\varpi_{\vartheta (i)}h_{\vartheta (i)}\right)}^{r_{i}}=\\ \langle \underset{\begin{array}{c} \mu_{\vartheta {\left(i\right)}_{l}}\in \Gamma_{h_{\vartheta (i)}}\\ i=1,2,\cdots n \end{array}}{\cup }\{\left[{\left(1-\prod_{\vartheta \in S_{n}}{\left(1-\prod_{i=1}^{n}{\left(1-{\left(1-{\underline{\mu }}_{\vartheta {\left(i\right)}_{l}}^{q}\right)}^{n\varpi_{\vartheta (i)}}\right)}^{r_{i}}\right)}^{1/n!}\right)}^{1/q}](\frac{\prod_{\vartheta \in S_{n}}\prod_{i=1}^{n}p_{\vartheta {\left(i\right)}_{l}}}{\prod_{\vartheta \in S_{n}}\prod_{i=1}^{n}\sum_{l=1}^{\left|\Gamma_{h_{\vartheta (i)}}\right|}p_{\vartheta {\left(i\right)}_{l}}}\right)\},\\ \underset{\begin{array}{c} v_{\vartheta {\left(i\right)}_{m}}\in \Psi_{h_{\vartheta (i)}}\\ i=1,2,\cdots n \end{array}}{\cup }\{\left[{\left(\prod_{\vartheta \in S_{n}}{\left(1-\prod_{i=1}^{n}{\left(1-\upsilon_{\vartheta {\left(i\right)}_{m}}^{n\varpi_{\vartheta (i)}}\right)}^{r_{i}}\right)}^{1/q}\right)}^{1/n!}](\frac{\prod_{\vartheta \in S_{n}}\prod_{i=1}^{n}{\bar{p}}_{\vartheta {\left(i\right)}_{m}}}{\prod_{\vartheta \in S_{n}}\prod_{i=1}^{n}\sum_{m=1}^{\left|\Psi_{h_{\vartheta (i)}}\right|}{\bar{p}}_{\vartheta {\left(i\right)}_{m}}}\right)\}\rangle \end{array},$$

and

$$\begin{array}{c} {\left(\frac{1}{n!}\underset{\vartheta \in S_{n}}{⊕}\overset{n}{\underset{i=1}{⊗}}{\left(n\varpi_{\vartheta (i)}h_{\vartheta (i)}\right)}^{r_{i}}\right)}^{\frac{1}{\sum_{j=1}^{n}r_{i}}}=\\ \langle \underset{\begin{array}{c} \mu_{\vartheta {\left(i\right)}_{l}}\in \Gamma_{h_{\vartheta (i)}}\\ i=1,2,\cdots n \end{array}}{\cup }\{\left[{\left({\left(1-\prod_{\vartheta \in S_{n}}{\left(1-\prod_{i=1}^{n}{\left(1-{\left(1-\mu_{\vartheta (i)}^{q}\right)}^{n\varpi_{\vartheta (i)}}\right)}^{r_{i}}\right)}^{1/n!}\right)}^{1/q}\right)}^{1/\sum_{i=1}^{n}r_{i}}](\frac{\prod_{\vartheta \in S_{n}}\prod_{i=1}^{n}p_{\vartheta {\left(i\right)}_{l}}}{\prod_{\vartheta \in S_{n}}\prod_{i=1}^{n}\sum_{l=1}^{\left|\Gamma_{h_{\vartheta (i)}}\right|}p_{\vartheta {\left(i\right)}_{l}}}\right)\},\\ \underset{\begin{array}{c} v_{\vartheta {\left(i\right)}_{m}}\in \Psi_{h_{\vartheta (i)}}\\ i=1,2,\cdots n \end{array}}{\cup }\{\left[{\left(1-{\left(1-\prod_{\vartheta \in S_{n}}{\left(1-\prod_{i=1}^{n}{\left(1-\upsilon_{\vartheta (i)}^{qn\varpi_{\vartheta (i)}}\right)}^{r_{i}}\right)}^{1/n!}\right)}^{1/\sum_{i=1}^{n}r_{i}}\right)}^{1/q}](\frac{\prod_{\vartheta \in S_{n}}\prod_{i=1}^{n}{\bar{p}}_{\vartheta {\left(i\right)}_{m}}}{\prod_{\vartheta \in S_{n}}\prod_{i=1}^{n}\sum_{m=1}^{\left|\Psi_{h_{\vartheta (i)}}\right|}{\bar{p}}_{\vartheta {\left(i\right)}_{m}}}\right)\}\rangle \end{array}.$$

Therefore, Theorem 2 is proved.

### 4.2 Some special form of the q-ROPHFPWMM operator

The q-ROPHFPWMM operator can integrate information more flexibly by using its special parameter vector, and describe the correlation relationship among any attributes. When the parameter vector *P* takes a specific value, the q-ROPHFPWMM operator will degenerates into other operators.

**Case 1.** When *P* = (1,0,⋯,0), q-ROPHFPWMM operator degenerates into q-rung orthopair probabilistic hesitant fuzzy power weight average (q-ROPHFPWA) operator:

$$\begin{array}{c} q-ROPHFPWMM^{\left(1,0,\cdots ,0\right)}(h_{1},h_{2},\cdots ,h_{n})=\frac{1}{n}\overset{n}{\underset{i=1}{⊕}}n\varpi_{i}h_{i}\\ =\langle \underset{\mu_{i_{l}}\in \Gamma_{h_{i}}}{\cup }\{\left[{\left(1-\prod_{i=1}^{n}{\left(1-\mu_{i_{l}}^{q}\right)}^{\varpi_{i}}\right)}^{1/q}](\frac{\prod_{i=1}^{n}p_{i_{l}}}{\prod_{i=1}^{n}\sum_{l=1}^{\left|\Gamma_{h_{i}}\right|}p_{i_{l}}}\right|\},\underset{v_{i_{m}}\in \Psi_{h_{i}}}{\cup }\{\left[\prod_{i=1}^{n}\upsilon_{i_{m}}^{\varpi_{i}}](\frac{\prod_{i=1}^{n}{\bar{p}}_{i_{m}}}{\prod_{i=1}^{n}\sum_{m=1}^{\left|\Psi_{h_{i}}\right|}{\bar{p}}_{i_{m}}}\right)\}\rangle \end{array}$$

**Case 2.** When *P* = (1,1,⋯,0), q-ROPHFPWMM operator degenerates into q-rung orthopair probabilistic hesitant fuzzy power weight Bonferroni mean (q-ROPHFPWBM) operator:

$$\begin{array}{c} q-ROPHFPWMM^{\left(1,1,0,\cdots ,0\right)}(h_{1},h_{2},\cdots ,h_{n})={\left(\frac{1}{n(n-1)}\overset{n}{\underset{\begin{array}{c} i,j=1\\ i\neq j \end{array}}{⊕}}(n\varpi_{i}h_{i}⊗n\varpi_{j}h_{j})\right)}^{\frac{1}{2}}\\ =\langle \underset{\begin{array}{l} \mu_{i_{l}}\in \Gamma_{h_{i}},\\ \mu_{j_{l}}\in \Gamma_{h_{j}} \end{array}}{\cup }\{\left[{\left(1-\prod_{i=1}^{n}{\left(1-(1-{\left(1-\mu_{i_{l}}^{q}\right)}^{n\varpi_{i}})(1-{\left(1-\mu_{j_{l}}^{q}\right)}^{n\varpi_{j}})\right)}^{\frac{1}{n(n-1)}}\right)}^{1/2q}](\frac{\prod_{\begin{array}{c} i,j=1\\ i\neq j \end{array}}^{n}p_{i_{l}}p_{j_{l}}}{\prod_{\begin{array}{c} i,j=1\\ i\neq j \end{array}}^{n}\sum_{l=1}^{\left|\Gamma_{h_{i}}\right|}p_{i_{l}}\sum_{l=1}^{\left|\Gamma_{h_{j}}\right|}p_{j_{l}}}\right)\},\\ \underset{v_{i_{m}}\in \Psi_{h_{i}}}{\cup }\{\left[{\left(1-{\left(1-\prod_{i=1}^{n}{\left(1-(1-\upsilon_{i_{m}}^{qn\varpi_{i}})(1-\upsilon_{j_{m}}^{qn\varpi_{j}})\right)}^{\frac{1}{n(n-1)}}\right)}^{\frac{1}{2}}\right)}^{1/q}](\frac{\prod_{\begin{array}{c} i,j=1\\ i\neq j \end{array}}^{n}{\bar{p}}_{i_{m}}{\bar{p}}_{j_{m}}}{\prod_{\begin{array}{c} i,j=1\\ i\neq j \end{array}}^{n}\sum_{l=1}^{\left|\Psi_{h_{i}}\right|}{\bar{p}}_{i_{m}}\sum_{l=1}^{\left|\Psi_{h_{j}}\right|}{\bar{p}}_{j_{m}}}\right)\}\rangle \end{array}$$

**Case 3.** When $P=(\overset{k}{\overset{︷}{1,1,\cdots ,1}},\overset{n-k}{\overset{︷}{0,\cdots ,0}})$, q-ROPHFPWMM operator degenerates into q-rung orthopair probabilistic hesitant fuzzy power weight Maclaurin symmetric mean (q-ROPHFPWMSM) operator:

$$\begin{array}{c} q-ROPHFPWMM^{P}(h_{1},h_{2},\cdots ,h_{n})={\left(\frac{1}{C_{n}^{k}}\underset{\vartheta^{\prime }\in {S^{\prime }}_{n}}{⊕}(\overset{k}{\underset{j=1}{⊗}}n\varpi_{\vartheta^{\prime }(j)}h_{\vartheta^{\prime }(j)})\right)}^{\frac{1}{k}}\\ =\langle \underset{\begin{array}{c} \mu_{\vartheta^{\prime }{\left(i\right)}_{l}}\in \Gamma_{h_{\vartheta^{\prime }(i)}}\\ i=1,2,\cdots ,k \end{array}}{\cup }\{\left[{\left({\left(1-\prod_{\vartheta^{\prime }\in {S^{\prime }}_{n}}{\left(1-\prod_{i=1}^{k}\left(1-{\left(1-\mu_{\vartheta^{\prime }{\left(i\right)}_{l}}^{q}\right)}^{n\varpi_{\vartheta^{\prime }(i)}}\right)\right)}^{1/C_{n}^{k}}\right)}^{1/q}\right)}^{1/k}](\frac{\prod_{\vartheta^{\prime }\in {S^{\prime }}_{n}}\prod_{i=1}^{k}p_{\vartheta^{\prime }{\left(i\right)}_{l}}}{\prod_{\vartheta^{\prime }\in {S^{\prime }}_{n}}\prod_{i=1}^{k}\sum_{l=1}^{\left|\Gamma_{h_{\vartheta^{\prime }(i)}}\right|}p_{\vartheta^{\prime }{\left(i\right)}_{l}}}\right)\},\\ \underset{\begin{array}{c} v_{\vartheta^{\prime }{\left(i\right)}_{m}}\in \Psi_{h_{\vartheta^{\prime }(i)}}\\ i=1,2,\cdots k \end{array}}{\cup }\{\left[{\left(1-{\left(1-\prod_{\vartheta^{\prime }\in {S^{\prime }}_{n}}{\left(1-\prod_{i=1}^{k}\left(1-\upsilon_{\vartheta^{\prime }{\left(i\right)}_{m}}^{qn\varpi_{\vartheta^{\prime }(i)}}\right)\right)}^{1/C_{n}^{k}}\right)}^{1/k}\right)}^{1/q}](\frac{\prod_{\vartheta^{\prime }\in {S^{\prime }}_{n}}\prod_{i=1}^{k}{\bar{p}}_{\vartheta^{\prime }{\left(i\right)}_{m}}}{\prod_{\vartheta^{\prime }\in {S^{\prime }}_{n}}\prod_{i=1}^{k}\sum_{m=1}^{\left|\Psi_{h_{\vartheta^{\prime }(i)}}\right|}{\bar{p}}_{\vartheta^{\prime }{\left(i\right)}_{m}}}\right)\}\rangle \end{array}$$

where *ϑ*′ = {*ϑ*′(1),*ϑ*′(2),⋯,*ϑ*′(*n*)} traversals all the k-tuple combination of (1,2,⋯,*n*), ${S^{\prime }}_{n}$ is the set of all *ϑ*′, and $C_{n}^{k}=\frac{n!}{k!(n-k)!}$ is the binomial coefficient.

### 4.3 Some properties of the q-ROPHFPMM operator

In this subsection, we discuss the properties of q-ROPHFPWMM operator, including idempotence, boundedness and monotonicity.

**Theorem 2. (Idempotency)** Let *h*_*i*_(*i* = 1,2,⋯,*n*) be a set of q-ROPHFE, if *h*_*i*_ = *h* for *h*_*i*_(*i* = 1,2,⋯,*n*), then

$$q-ROPHFPWMM^{P}(h_{1},h_{2},\cdots ,h_{n})=h$$

**Theorem 3. (Boundedness)** Let *h*_*i*_(*i* = 1,2,⋯,*n*) be a group of q-ROPHFE, *h*^+^ = max *h*_*i*_, *h*^−^ = min *h*_*i*_, then

$$h^{-}<q-ROPHFPWMM^{P}(h_{1},h_{2},\cdots ,h_{n})<h^{+}$$

**Theorem 4. (Monotonicity)** Let *h*_*i*_(*i* = 1,2,⋯,*n*), $h_{i}^{\prime }(i=1,2,\cdots ,n)$ be two groups of q-ROPHFE respectively, if $h_{i}<h_{i}^{\prime }$ for ∀*i*∈{1,2,⋯,*n*}, then

$$q-ROPHFPWMM^{P}(h_{1},h_{2},\cdots ,h_{n})<q-ROPHFPWMM^{P}(h_{1}^{\prime },h_{2}^{\prime },\cdots ,h_{n}^{\prime })$$

## 5. A novel MADM algorithm framework based on q-ROPHFPWMM operator

In this section, we construct the MADM algorithm framework based on the q-PHFPWMM operator. Let *A* = {*A*_1_,*A*_2_,⋯,*A*_*m*_} be the alternative set, *M* = {*M*_1_,*M*_2_,⋯,*M*_*n*_} be the attribute set for each scheme and ***ω*** = (*ω*_1_,*ω*_2_,…,*ω*_*n*_)^*T*^ be the weight vector of the attribute set *M*. The weight vector ***ω*** is used to represent the importance of different attributes in the decision-making process, where $\sum_{i=1}^{n}\omega_{i}=1$ and *ω*_*i*_∈[0,1]. The evaluation value of scheme *A*_*i*_ under attribute *M*_*j*_ is expressed by q-ROPHFE $h_{ij}^{\prime }$, and the evaluation values of all attributes are collected to form the q-rung orthopair probabilistic hesitant fuzzy decision matrix $H^{\prime }=(h_{ij}^{\prime }{)}_{m\times n}$. The steps of the MADM algorithm based on the q-ROPHFPWMM operator are as follows:

**Step1.** Construct an evaluation index system, collect evaluation information and transform it into a q-rung orthopair probabilistic hesitant fuzzy decision matrix.
**Step2.** The q-rung orthopair probabilistic hesitant fuzzy decision matrix is normalized to obtain the normalized q-rung orthopair probabilistic hesitant fuzzy decision matrix. The normalization is as follows:

$$h_{ij}=\{\begin{array}{l} h_{ij}^{\prime },\;M_{ij}\mathrm{is}\;\mathrm{the}\;\mathrm{benefit}\;\mathrm{attribute},\\ {\left(h_{ij}^{\prime }\right)}^{c},\;M_{ij}\mathrm{is}\;\mathrm{the}\;\mathrm{cost}\;\mathrm{attribute}. \end{array}.$$

**Step3.** Select the appropriate risk preference coefficient *ζ* according to the decision maker’s risk preference, and add elements to the set whose probability sum is less than 1 to make the probability sum equal to 1.
**Step4.** Calculate the support degree between *h*_*ij*_ and *h*_*ik*_ (*i* = 1,2,⋯*m*; *j*,*k* = 1,2,⋯*n* and *k*≠*l*.):

$$Sup(h_{ij},h_{ik})=1-d(h_{ij},h_{ik}),$$

wherein, *d*(*h*_*ij*_,*h*_*ik*_) is the distance between *h*_*ij*_ and *h*_*ik*_.

**Step5.** Calculate the syntheses support degree:

$$T(h_{ij})=\sum_{k=1,k\neq j}^{n}Sup(h_{ij},h_{ik})$$

**Step6.** Calculate the power weight *σ*_*ij*_ of *h*_*ij*_:

$$\sigma_{ij}=\frac{\omega_{j}(1+T(h_{ij}))}{\sum_{j=1}^{n}\omega_{j}(1+T(h_{ij}))}.$$

**Step7.** Use the q-PHFPWMM operator to aggregate the evaluation values *h*_*ij*_ of the scheme *A*_*i*_ under different attributes *M*_*j*_ to obtain the comprehensive evaluation value *h*_*i*_:

$$h_{i}=q-PHFPWMM(h_{i1},h_{i2},\cdots ,h_{in})$$

**Step8.** Calculate the score function *S*(*h*_*i*_) and deviation degree *D*(*h*_*i*_) of the comprehensive evaluation value *h*_*i*_.
**Step9.** Sort the candidate solutions according to their score and deviation degree, and select the corresponding optimal solution.

## 6. The case on site selection assessment for landfill

### 6.1 Influencing factors of landfill site selection

With the advancement of urbanization, the scale of cities continues to expand, and the urban population soars, resulting in more and more garbage generated in the city. In recent years, the problem of garbage disposal has emerged in many cities around the world. At present, the main methods of urban waste disposal are through incineration for power generation and sanitary landfill. However, the way of incineration for power generation is not accepted by most citizens. Therefore, most cities choose to build new landfills to reduce the burden of waste disposal, but the site selection needs to consider several factors, including:

1. Geographic location
  First of all, the site selection of the landfill site should consider whether its geographic location is consistent with the overall planning of the city [42]. Secondly, it also needs to consider its radiative capacity, i.e. the range of services it can provide. On the premise of reducing the burden of the city, it can also share part of the burden of the surrounding cities.
2. Operating cost
  Operating costs of landfill sites mainly come from four aspects, including land utilization, equipment maintenance, garbage transportation and manpower [43]. Among them, the cost of land resources is huge. Landfills need a large amount of land resources, and the landfill can only be used for greening after the landfill is filled with green. And the land is not regenerative for 100 years.
3. Traffic conditions
  Landfills are typically located in suburban areas, away from densely populated areas. In order to reduce the transportation cost of garbage, the distance between the landfill site and the urban area and the road conditions should be considered [44]. In addition, because of the "NIMBY" effect of garbage trucks, cities often set garbage truck driving hours between 3 a.m. and 5 a.m.
4. Environmental pollution
  Landfills inevitably cause pollution to the surrounding environment, mainly including air pollution, soil pollution and water pollution [45]. Air pollution mainly comes from waste gas, dust and inhalable particles released from garbage disposal process, which in turn leads to acid rain and smog. In addition, garbage rotting released harmful gases, such as hydrogen sulfide, can also seriously pollute the atmosphere. Soil pollution is caused by the fact that heavy metals, chemical agents and plastic products contained in garbage cannot be degraded in the soil, which leads to the decrease of crop production and quality in surrounding farmland. In the process of garbage stacking and corruption, a large amount of acidic and alkaline organic pollutants will be generated, which will dissolve heavy metals in the garbage. These harmful components will flow into the river water after being washed by rainwater and cause surface water pollution. At the same time, the leachate from the garbage seeps into the soil and causes groundwater pollution.

### 6.2 Evaluation process of landfill site selection

There are 5 addresses *A*_*i*_(*i* = 1,2,3,4,5) as candidate sites for landfill construction, and professionals will evaluate the candidate sites based on four indicators such as geographical location (*M*_1_), operating cost (*M*_2_), traffic condition (*M*_3_) and environmental pollution (*M*_4_). Since different indicators have different influences on the comprehensive evaluation of candidate addresses, let the weight vector ***ω*** = (0.2,0.3,0.1,0.4)^*T*^.

**Fig 1:**
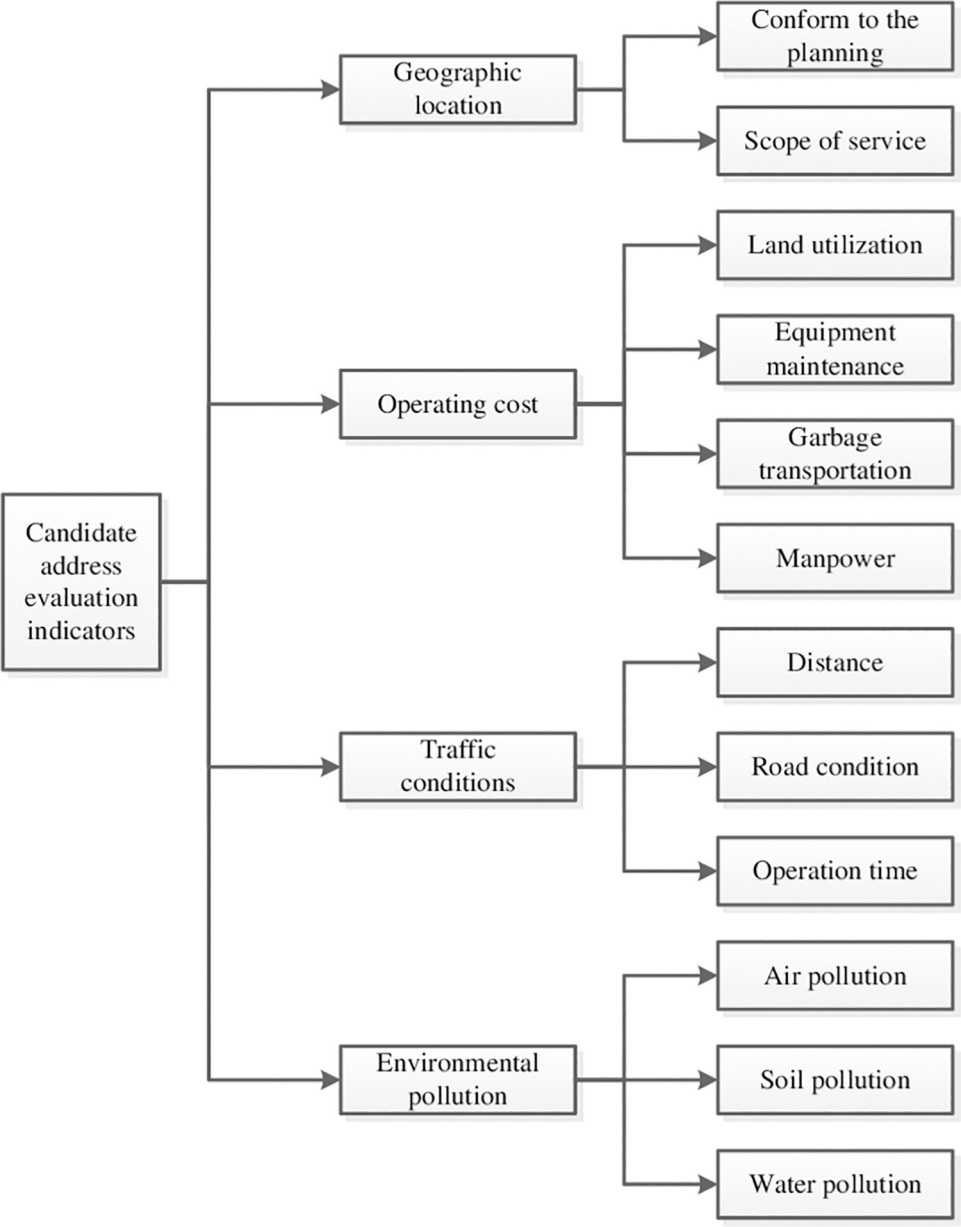
The evaluation index system of candidate addresses.

**Step 1.** The influential factors of landfill site selection are analyzed to build an evaluation index system, as shown in Fig 1.

The evaluation information was collected and transformed into a q-rung orthopair probabilistic hesitant fuzzy decision matrix (*q*≥3), as shown in Table 1. Among them, *M*_1_ and *M*_3_ are benefit attribute indexes, *M*_2_ and *M*_4_ are cost attribute indexes. And we can’t intuitively know from the data in Table 1 which candidate address is better.

**Table 1:** The q-rung orthopair probabilistic hesitant fuzzy decision matrix.

Candidate address	Evaluation index			
	*M*_1_	*M*_2_	*M*_3_	*M*_4_
*A*_1_	<{0.7|0.6,0.6|0.4}, {0.5|0.6,0.4|0.2}>	<{0.4|0.5,0.3|0.3}, {0.8|0.2,0.7|0.8}>	<{0.3|0.7,0.2|0.3}, {0.8|0.2,0.7|0.6}>	<{0.5|0.4,0.4|0.4}, {0.7|0.6,0.6|0.4}>
*A*_2_	<{0.5|0.8,0.4|0.2}, {0.6|0.1,0.5|0.9}>	<{0.8|0.6,0.7|0.3}, {0.7|0.2,0.6|0.8}>	<{0.4|0.5,0.3|0.4}, {0.8|0.4,0.7|0.6}>	<{0.6|0.3,0.5|0.7}, {0.5|0.6,0.4|0.4}>
*A*_3_	<{0.8|0.7,0.7|0.3}, {0.4|0.3,0.3|0.5}>	<{0.3|0.5,0.2|0.5}, {0.9|0.4,0.8|0.6}>	<{0.7|0.4,0.6|0.5}, {0.8|0.2,0.7|0.6}>	<{0.3|0.6,0.2|0.4}, {0.6|0.8,0.5|0.2}>
*A*_4_	<{0.4|0.5,0.3|0.3}, {0.8|0.2,0.7|0.6}>	<{0.6|0.7,0.5|0.1}, {0.6|0.3,0.5|0.5}>	<{0.6|0.5,0.5|0.5}, {0.9|0.4,0.8|0.4}>	<{0.7|0.6,0.6|0.4}, {0.6|0.2,0.5|0.8}>
*A*_5_	<{0.7|0.4,0.6|0.4}, {0.8|0.8,0.7|0.2}>	<{0.5|0.6,0.4|0.4}, {0.7|0.3,0.6|0.7}>	<{0.8|0.6,0.7|0.4}, {0.6|0.1,0.5|0.9}>	<{0.4|0.6,0.3|0.4}, {0.8|0.2,0.7|0.6}>

**Step 2.** Standardize the q-rung orthopair probabilistic hesitant fuzzy decision matrix in Step1 to obtain the standardized q-rung orthopair probabilistic hesitant fuzzy decision matrix.
**Step 3.** Assume that the decision-maker is risk averse, so take the risk preference coefficient *ζ* = 0. Add elements to the set whose probability sum is less than 1 in the normalized q-rung orthopair probabilistic hesitant fuzzy decision matrix so that the probability sum is equal to 1.
**Step 4.** Calculate the support degree *Sup*(*h*_*ij*_,*h*_*ik*_) between *h*_*ij*_ and *h*_*ik*_, where *i* = 1,2,3,4,5, *k*,*l* = 1,2,3,4 and *k*≠*l*. For convenience, let $S_{j}^{k}=(Sup(h_{1j},h_{1k}),Sup(h_{2j},h_{2k}),\cdots Sup(h_{ij},h_{ik}))$.

$S_{1}^{2}=S_{2}^{1}=(0.7381,0.7536,0.4944,0.8117,0.8027)$, $S_{1}^{3}=S_{3}^{1}=(0.7264,0.7988,0.7238,0.8198,0.7262)$,

$S_{1}^{4}=S_{4}^{1}=(0.8022,0.9634,0.6678,0.7598,0.9302)$, $S_{2}^{3}=S_{3}^{2}=(0.9829,0.6936,0.7640,0.6840,0.7425)$,

$S_{2}^{4}=S_{4}^{2}=(0.9329,0.7707,0.7164,0.9625,0.9059)$, $S_{3}^{4}=S_{4}^{3}=(0.9236,0.7682,0.7841,0.6665,0.6721)$.

**Step 5.** Calculate the syntheses support degree *T*(*h*_*ij*_) and get matrix *T* = (*T*(*h*_*ij*_))_5×4_:

$$T=[\begin{array}{cccc} 2.2667 & 2.6539 & 2.6329 & 2.6587\\ 2.5158 & 2.2179 & 2.2606 & 2.5023\\ 1.8860 & 1.9748 & 2.2719 & 2.1683\\ 2.3913 & 2.4222 & 2.1703 & 2.3528\\ 2.3591 & 2.4511 & 2.1408 & 2.4082 \end{array}].$$

**Step 6.** Calculate the power weight of *h*_*ij*_ and get the matrix *σ* = (*σ*_*ij*_)_5×4_:

$$\sigma =[\begin{array}{cccc} 0.1827 & 0.3065 & 0.1016 & 0.4092\\ 0.2071 & 0.2843 & 0.0960 & 0.4126\\ 0.1884 & 0.2913 & 0.1068 & 0.4136\\ 0.2017 & 0.3053 & 0.0943 & 0.3988\\ 0.1985 & 0.3059 & 0.0928 & 0.4028 \end{array}].$$

**Step 7.** Utilize the q-PHFPWMM operator (*q* = 3 and *P* = (1,1,0,0)) to aggregate the evaluation information of each candidate address to get its comprehensive evaluation value *h*_*i*_(*i* = 1,2,3,4,5). The results are shown in Table 2.

**Table 2:** The comprehensive evaluation value.

*h*_1_	<{0.3236|0.1312, 0.2995|0.1312, 0.3213|0.0562, 0.2967|0.0562, 0.3104|0.0787, 0.2835|0.0787, 0.3078|0.0337, 0.2802|0.0337, 0.3023|0.0875, 0.2788|0.0875, 0.2997|0.0375, 0.2759|0.0375, 0.2879|0.0525, 0.2626|0.0525, 0.2849|0.0225, 0.2590|0.0225}, {0.9196|0.0225, 0.9102|0.0150, 0.9151|0.0675, 0.9055|0.0450, 0.9096|0.0900, 0.8995|0.0600, 0.9051|0.2700, 0.8948|0.1800, 0.9124|0.0075, 0.9022|0.0050, 0.9078|0.0225, 0.8974|0.0150, 0.9020|0.0300, 0.8912|0.0200, 0.8974|0.0900, 0.8864|0.0600}>
*h*_2_	<{0.4088|0.8890, 0.3812|0.2074, 0.4057|0.0711, 0.3774|0.1659, 0.3808|0.0444, 0.3547|0.1037, 0.3775|0.0355, 0.3508|0.0829, 0.3995|0.0222, 0.3696|0.0519, 0.3962|0.0178, 0.3654|0.0415, 0.3709|0.0111, 0.3430|0.0259, 0.3673|0.0089, 0.3386|0.0207} {0.8987|0.0048, 0.8907|0.0032, 0.8938|0.0072, 0.8857|0.0048, 0.8877|0.0192, 0.8787|0.0128, 0.8828|0.0288, 0.8737|0.0192, 0.8902|0.0432, 0.8815|0.0288, 0.8853|0.0648, 0.8766|0.0432, 0.8790|0.1728, 0.8695|0.1152, 0.8741|0.2592, 0.8645|0.1728}>
*h*_3_	<{0.3348|0.0933, 0.3251|0.0622, 0.3159|0.1167, 0.3041|0.0778, 0.3275|0.0933, 0.3171|0.0622, 0.3071|0.1167, 0.2938|0.0778, 0.3111|0.0800, 0.3010|0.0533, 0.2932|0.1000, 0.2814|0.0667, 0.3035|0.0800, 0.2926|0.0533, 0.2843|0.1000, 0.2710|0.0667}, {0.9136|0.0300, 0.9047|0.0075, 0.9089|0.0900, 0.8997|0.0225, 0.9022|0.0450, 0.8927|0.0113, 0.8974|0.1350, 0.8878|0.0337, 0.9052|0.0500, 0.8950|0.0125, 0.9004|0.1500, 0.8901|0.0375, 0.8935|0.0750, 0.8829|0.0188, 0.8887|0.2250, 0.8870|0.0563}>
*h*_4_	<{0.3867|0.1641, 0.3606|0.1094, 0.3777|0.1641, 0.3511|0.1094, 0.3636|0.0234, 0.3384|0.0156, 0.3527|0.0234, 0.3275|0.0156, 0.4089|0.0984, 0.3839|0.0656, 0.4013|0.0984, 0.3758|0.0656, 0.3892|0.0141, 0.3643|0.0094, 0.3805|0.0141, 0.3553|0.0094} {0.9172|0.0094, 0.9085|0.0375, 0.9127|0.0094, 0.9039|0.0375, 0.9079|0.0156, 0.8979|0.0625, 0.9034|0.0156, 0.8933|0.0625, 0.9095|0.0281, 0.9005|0.1125, 0.9051|0.0281, 0.8959|0.1125, 0.9001|0.0469, 0.8898|0.1875, 0.8956|0.0469, 0.8852|0.1875}>
*h*_5_	<{0.3720|0.1080, 0.3599|0.0720, 0.3544|0.0720, 0.3412|0.0480, 0.3720|0.0720, 0.3599|0.0480, 0.3544|0.0480, 0.3412|0.0320, 0.3536|0.1080, 0.3404|0.0720, 0.3357|0.0720, 0.3216|0.0480, 0.3536|0.0720, 0.3404|0.0480, 0.3357|0.0480, 0.3216|0.0320}, {0.9318|0.0060, 0.9217|0.0180, 0.9270|0.0540, 0.9166|0.1620, 0.9240|0.0140, 0.9129|0.0420, 0.9189|0.1260, 0.9076|0.3780, 0.9251|0.0015, 0.9147|0.0045, 0.9202|0.0135, 0.9096|0.0405, 0.9170|0.0035, 0.9057|0.0105, 0.9118|0.0315, 0.9003|0.0945}>

**Step 8.** Calculate the score function *S*(*h*_*i*_) of each candidate address: *S*(*h*_1_) = 0.4763, *S*(*h*_2_) = 0.4289, *S*(*h*_3_) = 0.4845, *S*(*h*_4_) = 0.4187, *S*(*h*_5_) = 0.4626.
**Step 9.** Sort the candidate address according to the score value of the candidate address to get *A*_3_≻*A*_1_≻*A*_5_≻*A*_2_≻*A*_4_. Therefore, the best landfill site is A.

### 6.3 Parameter analysis

By changing the value of the parameter vector *P*, the ranking results of different landfills are obtained by using the q-ROPHFPWMM operator (*q* = 3), as shown in Table 3.

**Table 3:** 

The score value and ranking result with different values of parameter vector *P*.		
Parameter vector *P*	*S*(*h*_*i*_)(*i* = 1,2,3,4,5)	Ranking
*P* = (1,0,0,0)	***S***(***h***_**1**_) = 0.4776,***S***(***h***_**2**_) = 0.4534,***S***(***h***_**3**_) = 0.5002,***S***(***h***_**4**_) = 0.4627,***S***(***h***_**5**_) = 0.4870	*A*_3_≻*A*_5_≻*A*_1_≻*A*_4_≻*A*_2_
*P* = (1,1,0,0)	***S***(***h***_**1**_) = 0.4763,***S***(***h***_**2**_) = 0.4289,***S***(***h***_**3**_) = 0.4845,***S***(***h***_**4**_) = 0.4187,***S***(***h***_**5**_) = 0.4626	*A*_3_≻*A*_1_≻*A*_5_≻*A*_2_≻*A*_4_
*P* = (1,2,0,0)	***S***(***h***_**1**_) = 0.4767,***S***(***h***_**2**_) = 0.4302,***S***(***h***_**3**_) = 0.4857,***S***(***h***_**4**_) = 0.4201,***S***(***h***_**5**_) = 0.4628	*A*_3_≻*A*_1_≻*A*_5_≻*A*_2_≻*A*_4_
*P* = (1,3,0,0)	***S***(***h***_**1**_) = 0.4771,***S***(***h***_**2**_) = 0.4317,***S***(***h***_**3**_) = 0.4868,***S***(***h***_**4**_) = 0.4215,***S***(***h***_**5**_) = 0.4631	*A*_3_≻*A*_1_≻*A*_5_≻*A*_2_≻*A*_4_
*P* = (1,1,1,0)	***S***(***h***_**1**_) = 0.4806,***S***(***h***_**2**_) = 0.4412,***S***(***h***_**3**_) = 0.4892,***S***(***h***_**4**_) = 0.4294,***S***(***h***_**5**_) = 0.4698	*A*_3_≻*A*_1_≻*A*_5_≻*A*_2_≻*A*_4_
*P* = (1,2,1,0)	***S***(***h***_**1**_) = 0.4817,***S***(***h***_**2**_) = 0.4438,***S***(***h***_**3**_) = 0.4915,***S***(***h***_**4**_) = 0.4319,***S***(***h***_**5**_) = 0.4714	*A*_3_≻*A*_1_≻*A*_5_≻*A*_2_≻*A*_4_
*P* = (1,2,2,0)	***S***(***h***_**1**_) = 0.4823,***S***(***h***_**2**_) = 0.4455,***S***(***h***_**3**_) = 0.4928,***S***(***h***_**4**_) = 0.4336,***S***(***h***_**5**_) = 0.4724	*A*_3_≻*A*_1_≻*A*_5_≻*A*_2_≻*A*_4_
*P* = (1,1,1,1)	***S***(***h***_**1**_) = 0.4831,***S***(***h***_**2**_) = 0.4487,***S***(***h***_**3**_) = 0.5106,***S***(***h***_**4**_) = 0.4491,***S***(***h***_**5**_) = 0.4806	*A*_3_≻*A*_1_≻*A*_5_≻*A*_4_≻*A*_2_

The score value and ranking result with different values of parameter vector *q*.		
Parameter *q*	*S*(*h*_*i*_)(*i* = 1,2,3,4,5)	Ranking
*q* = 3	***S***(***h***_**1**_) = 0.4763,***S***(***h***_**2**_) = 0.4289,***S***(***h***_**3**_) = 0.4845,***S***(***h***_**4**_) = 0.4187,***S***(***h***_**5**_) = 0.4626	*A*_3_≻*A*_1_≻*A*_5_≻*A*_2_≻*A*_4_
*q* = 5	***S***(***h***_**1**_) = 0.4770,***S***(***h***_**2**_) = 0.4292,***S***(***h***_**3**_) = 0.4877,***S***(***h***_**4**_) = 0.4186,***S***(***h***_**5**_) = 0.4638	*A*_3_≻*A*_1_≻*A*_5_≻*A*_2_≻*A*_4_
*q* = 7	***S***(***h***_**1**_) = 0.4774,***S***(***h***_**2**_) = 0.4293,***S***(***h***_**3**_) = 0.4896,***S***(***h***_**4**_) = 0.4185,***S***(***h***_**5**_) = 0.4646	*A*_3_≻*A*_1_≻*A*_5_≻*A*_2_≻*A*_4_
*q* = 10	***S***(***h***_**1**_) = 0.4777,***S***(***h***_**2**_) = 0.4292,***S***(***h***_**3**_) = 0.4913,***S***(***h***_**4**_) = 0.4182,***S***(***h***_**5**_) = 0.4653	*A*_3_≻*A*_1_≻*A*_5_≻*A*_2_≻*A*_4_
*q* = 20	***S***(***h***_**1**_) = 0.4780,***S***(***h***_**2**_) = 0.4286,***S***(***h***_**3**_) = 0.4934,***S***(***h***_**4**_) = 0.4174,***S***(***h***_**5**_) = 0.4662	*A*_3_≻*A*_1_≻*A*_5_≻*A*_2_≻*A*_4_
*q* = 30	***S***(***h***_**1**_) = 0.4729,***S***(***h***_**2**_) = 0.4283,***S***(***h***_**3**_) = 0.4942,***S***(***h***_**4**_) = 0.4170,***S***(***h***_**5**_) = 0.4665	*A*_3_≻*A*_1_≻*A*_5_≻*A*_2_≻*A*_4_
*q* = 50	***S***(***h***_**1**_) = 0.4728,***S***(***h***_**2**_) = 0.4220,***S***(***h***_**3**_) = 0.4744,***S***(***h***_**4**_) = 0.4137,***S***(***h***_**5**_) = 0.4569	*A*_3_≻*A*_1_≻*A*_5_≻*A*_2_≻*A*_4_
*q* = 70	***S***(***h***_**1**_) = 0.4727,***S***(***h***_**2**_) = 0.4217,***S***(***h***_**3**_) = 0.4743,***S***(***h***_**4**_) = 0.4134,***S***(***h***_**5**_) = 0.4568	*A*_3_≻*A*_1_≻*A*_5_≻*A*_2_≻*A*_4_
*q* = 100	***S***(***h***_**1**_) = 0.4726,***S***(***h***_**2**_) = 0.4216,***S***(***h***_**3**_) = 0.4742,***S***(***h***_**4**_) = 0.4132,***S***(***h***_**5**_) = 0.4567	*A*_3_≻*A*_1_≻*A*_5_≻*A*_2_≻*A*_4_

It can be seen from Table 3:

1. The optimal candidate addresses under different parameter vectors are all x, which indicates that the multi-attribute decision making method based on q-ROPHFPWMM operator is effective and reliable.
2. As can be seen from Table 1, candidate address *A*_3_ has the smallest membership degree and the largest non-membership degree under the evaluation index *M*_2_. However, the order of *A*_3_ is not affected by this, resulting in a lower order, which shows that the PA operator plays a good role.
3. When *P* = (1,0,0,0), the ranking result is *A*_3_≻*A*_5_≻*A*_1_≻*A*_4_≻*A*_2_. It is quite different from the ranking result when the parameter vector selects other values. Only the best candidate addresses are the same. Because q-ROPHFPWMM operator degenerates into q-ROPHFPWA operator, when the parameter vector *P* = (1,0,0,0). And the q-ROPHFPWA operator cannot reflect the correlation relationship between attributes in the process of information aggregation.
4. When the correlation between attributes is considered, that is, when the non-zero values in vector *P* are not less than 2, the ranking results obtained by q-ROPHFPWMM operator are basically the same, which indicates that q-ROPHFPWMM operator has good robustness.

The number of non-zero values in the parameter vector *P* is the number of attributes that have an association relationship. For example, when the parameter vector *P* = (1,1,0,0), the q-ROPHFPWMM operator can describe the relationship between any two attributes. The decision maker can determine the number of non-zero values according to the correlation between evaluation indexes in practical problems. In addition, the decision-maker can also choose the size of the non-zero value according to his decision preference and risk attitude.

Next, the influence of parameter *q* on the score function and the ranking result is further analyzed. Take the parameter vector *P* = (1,1,0,0) and assign different values to the parameter *q*. The results are shown in Table 4.

**Table 4:** The score value and ranking results based on different operators.

Operator	*S*(*h*_*i*_)(*i* = 1,2,3,4,5)	Ranking
The method by the q-RDHFWHM operator [46]	***S***(***h***_**1**_) = 0.2258,***S***(***h***_**2**_) = 0.2901,***S***(***h***_**3**_) = 0.2500,***S***(***h***_**4**_) = 0.2778,***S***(***h***_**5**_) = 0.2420	*A*_2_≻*A*_4_≻*A*_3_≻*A*_5_≻*A*_1_
The method by the DHq-ROFWMM operator [47]	***S***(***h***_**1**_) = 0.1881,***S***(***h***_**2**_) = 0.2373,***S***(***h***_**3**_) = 0.1989,***S***(***h***_**4**_) = 0.2357,***S***(***h***_**5**_) = 0.2115	*A*_2_≻*A*_4_≻*A*_5_≻*A*_3_≻*A*_1_
The method by the q-ROHFWPGHM operator [48]	***S***(***h***_**1**_) = 0.2190,***S***(***h***_**2**_) = 0.2664,***S***(***h***_**3**_) = 0.2462,***S***(***h***_**4**_) = 0.2783,***S***(***h***_**5**_) = 0.2402	*A*_4_≻*A*_2_≻*A*_3_≻*A*_5_≻*A*_1_
The method by the q-RPDHFPWMM operator [49]	***S***(***h***_**1**_) = 0.5000,***S***(***h***_**2**_) = 0.5000,***S***(***h***_**3**_) = 0.5000,***S***(***h***_**4**_) = 0.5000,***S***(***h***_**5**_) = 0.5000	Indistinguishable

When the parameter *q* changes, the score of the candidate address first increases and then decreases with the increase of *q*, and finally tends to be stable. At the same time, the sorting result of candidate addresses has no change. But when q is greater than 50, the support between *h*_*ij*_ and *h*_*ik*_ tends to 1, and the power average operator loses its function. However, when *q* is greater than 50, the support degree *Sup*(*h*_*ij*_,*h*_*ik*_) between *h*_*ij*_ and *h*_*ik*_ tends to 1, making PA operator useless. Thus, the selection of the value of *q* is very important. Combined with the discussion and analysis of the parameter *q* of q-RDHFWHM operator in reference [46], the optimal principle for selecting the value of *q* is given. The principle is that the value of *q* should be the smallest positive integer that satisfies the sum of the qth-power of the maximum values in the membership and non-membership degrees less than 1.

### 6.4 Comparative analysis

The most important advantage of q-ROPHFS is that it uses probability to represent the decision maker’s hesitation, and it reduces the loss of information by normalizing the probability in the process of information integration. In order to highlight the advantages of q-ROPHFPWMM operator, a comparative analysis was made with the operators mentioned in reference [46–49], and the ranking results are shown in Table 4.

The ranking results based on q-RDHFWHM operator, DHq-ROFWMM operator and q-ROHFWPGHM operator are quite different from the sorting results based on the operator mentioned in this paper. This is because the operators proposed in reference [46–48] do not consider probability and fail to comprehensively describe the hesitations of decision makers in the process of information integration. They lost some information in the process of information integration.

The q-RPDHFPMM operator proposed in reference [49] uses probability to describe the hesitations of decision makers, but information loss also occurs in the process of information integration. This is because the probability of q-RPDHFPMM operator has not been normalized in the process of information integration, leading to a small probability in the comprehensive evaluation value, almost zero. Furthermore, the score values of candidate addresses are similar, and it is impossible to distinguish which candidate address is better. In addition, when there are too many operations or too many data in the process of information integration, the probability of the result obtained after operation tends to zero more easily. Compared with the operator proposed in this paper, its application scope is smaller.

## 7. Conclusion and future studies

Garbage disposal is an important part of urban governance, and the construction of new landfill sites is an important way to solve the environmental pollution caused by the lack of garbage disposal capacity. The construction of landfill needs to coordinate its interests with the surrounding environment, residents and government, etc. Scientific and reasonable site selection method can maximize the interests. The site selection of landfill sites is affected by many factors, and people’s cognitive ability is limited, so it is impossible to give an accurate evaluation value. At present, the evaluation method of landfill site selection scheme based on FS and IFS has effectively solved this problem [39,40], but it does not take into account the hesitation of decision makers. Therefore, in order to better solve the indecisiveness of decision-makers in the decision-making process, q-ROPHFS is proposed and the MADM algorithm based on q-ROPHFPWMM operator is constructed.

The MADM algorithm presented in this paper is applied to a practical case of landfill site selection evaluation, and the applicability and rationality of the algorithm are illustrated. The location model based on the q-PHFPWMM operator get the best candidate for the landfill site address is unique, which indicates that the model is reliable and effective. Then, the parameter vector *P* in the model is adjusted to analyze its influence on the results. When there is only one non-zero value in the parameter vector *P*, the correlation between evaluation factors cannot be captured, so the ranking results differ greatly. However, no matter how the parameter vector *P* is valued, the optimal candidate address is the same, which indicates that this location model has good robustness. By comparing the ranking results of parameter vectors with different values, it can be seen that q-ROPHFPWMM operator has significant advantages in describing the correlation between evaluation attributes and eliminating the influence of unreasonable values. In addition, combined with the operator mentioned in the reference [46–49], the biggest advantage of the MADM algorithm based on q-ROPHFPWMM operator is that it comprehensively represents the hesitations of decision-makers in the decision-making process, and reduces information loss in the process of information integration.

Through the case of landfill site evaluation, we can know that the algorithm has significant advantages. It converts the evaluation information into q-ROPHFE, effectively reduces the loss of information and makes the decision results more reliable. Moreover, q-ROPHFS allows the probability sum of the elements in the set to be less than 1, which provides more hesitation space for decision makers to give a more reasonable evaluation. In addition, q-ROPHFS allow the sum of membership and non-membership to be greater than 1 and their sum to the q power to be less than 1. It greatly expands the value range of evaluation information and improves the freedom of decision makers. The q-ROPHFPWMM operator used in the algorithm includes the advantages of both power average operator and Muirhead mean operator. Therefore, it effectively reduces the influence of unreasonable evaluation information given by decision makers on the results, and describes the correlation between any evaluation factors by changing the number of non-zero values of the parameter vector, and the decision makers can also choose the size of non-zero values according to their decision preferences.

In conclusion, the work of this paper is summarized as follows. Aiming at the problem that q-order hesitant fuzzy sets cannot fully describe the indecisiveness of decision-makers in the decision-making process, we creatively introduce probability into q-rung orthopair hesitant fuzzy sets, and proposes q-rung orthopair probabilistic hesitant fuzzy sets. Furthermore, the operation of q-ROPHFE is defined. The scoring function and deviation degree are given. The distance measure of q-ROPHFE is defined, and three calculation formulas are given. In order to eliminate the influence of extreme values and the correlation between attributes, the PA operator and Muirhead mean operator are combined and extended to the q-rung orthopair probabilistic hesitant fuzzy set, and the q-rung orthopair probabilistic hesitant fuzzy power weight Muirhead mean operator is proposed. Then, a novel multi-attribute decision-making algorithm based on q-ROPHFPWMM operator is proposed. This algorithm is used to solve the problem of landfill site selection.

In the future, we can study the Einstein geometric aggregation operator based on q-ROPHFE operation and distance measure [50]. At the same time, the operation of q-ROPHFE can be improved by combining T norm and T co-norm, and the distance measure can also be improved. In addition, the power Muirhead average operator can also be extended to complex q-rung orthopair fuzzy set [51], q-rung orthopair m-polar fuzzy set [52] and spherical and T-spherical fuzzy set [53], and its special properties under different environments are discussed.

## References

[1] Kurniawan TA, Lo WH, Singh D, Othman MHD, Avtar R, Hwang GH, et al. A societal transition of MSWM in Xiamen (China) toward a circular economy through integrated waste recycling and technological digitization. Environmental Pollution. 2021; 277: 116741. doi: 10.1016/j.envpol.2021.116741 33652179

[2] Song JB, Sun Y, Jin LL. PESTEL analysis of the development of the waste-to-energy incineration industry in China. Renewable and Sustainable Energy Reviews. 2017; 80: 276–289.

[3] Liu Y, Huang JK. Rural domestic waste disposal: an empirical analysis in five provinces of China. China Agricultural Economic Review. 2014; 6(4): 558–573.

[4] Eiselt HA, Marianov V. Location modeling for municipal solid waste facilities. Computers & Operations Research. 2014; 62: 305–315.

[5] Kim KR, Owens G. Potential for enhanced phytoremediation of landfills using biosolids—a review. Journal of Environmental Management. 2010; 91(4): 791–797. doi: 10.1016/j.jenvman.2009.10.017 19939550

[6] Abad PMS, Pazira E, Abadi MHM, Abdinezhad P. Application AHP-PROMETHEE technic for landfill site selection on based assessment of aquifers vulnerability to pollution. Iranian Journal of Science and Technology-Transactions of Civil Engineering. 2021; 45(2): 1011–1030.

[7] Njoku PO, Edokpayi JN, Odiyo JO. Health and environmental risks of residents living close to a landfill: a case study of thohoyandou landfill, Limpopo Province, South Africa. International Journal of Environmental Research and Public Health. 2019; 16(12): 2125. doi: 10.3390/ijerph16122125 31208082 PMC6617357

[8] Yang ZL, Chang JP. A multi-attribute decision-making-based site selection assessment algorithm for garbage disposal plant using interval q-rung orthopair fuzzy power Muirhead mean operator. Environmental Research. 2021; 193: 110385. doi: 10.1016/j.envres.2020.110385 33166534

[9] Jiang S, Li ZG, Gao C. Study on site selection of municipal solid waste incineration plant based on swarm optimization algorithm. Waste Management & Research. 2020; 0734242X20981619. 10.1177/0734242X20981619 33357101

[10] Zarin R, Azmat M, Naqvi SR, Saddique Q, Ullah S. Landfill site selection by integrating fuzzy logic, AHP, and WLC method based on multi-criteria decision analysis. Environmental Science and Pollution Research. 2021; 28(16): 19726–19741. doi: 10.1007/s11356-020-11975-7 33410005

[11] Rezaeisabzevar Y, Bazargan A, Zohourian, B. Landfill site selection using multi criteria decision making: Influential factors for comparing locations. Journal of Environmental Sciences. 2020; 93: 170–184. 10.1016/j.jes.2020.02.030 32446453

[12] Eghtesadifard M, Afkhami P, Bazyar A. An integrated approach to the selection of municipal solid waste landfills through GIS, K-Means and multi-criteria decision analysis. Environmental Research. 2020; 185: 109348. doi: 10.1016/j.envres.2020.109348 32278923

[13] Zadeh LA. Fuzzy sets. Information & Control. 1965; 8(3): 338–356.

[14] Atanassov K. Intuitionistic fuzzy sets. Fuzzy Sets and Systems. 1986; 20(1): 87–96.

[15] Turksen IB. Interval valued fuzzy sets based on normal forms. Fuzzy Sets and Systems. 1986; 20(2): 191–210.

[16] Torra V. Hesitant fuzzy sets. International Journal of Intelligent Systems. 2010; 25(6): 529–539.

[17] Yager RR. Pythagorean membership grades in multicriteria decision making. IEEE Transactions on Fuzzy Systems. 2014; 22(4): 958–965.

[18] Yager RR. Generalized orthopair fuzzy sets. IEEE Transactions on Fuzzy Systems. 2017; 25(5): 1222–1230.

[19] Liu DH, Peng D, Liu ZM. The distance measures between q-rung orthopair hesitant fuzzy sets and their application in multiple criteria decision making. International Journal of Intelligent Systems. 2019; 34(9): 2104–2121.

[20] Bedregal B, Beliakov G, Bustince H, Calvo T, Mesiar R, Paternain D. A class of fuzzy multisets with a fixed number of memberships. Information Sciences. 2012; 189: 1–17.

[21] Xu ZS, Zhou W. Consensus building with a group of decision makers under the hesitant probabilistic fuzzy environment. Fuzzy Optimization and Decision Making. 2017; 16(4): 481–503.

[22] Zhang S, Xu ZS, He Y. Operations and integrations of probabilistic hesitant fuzzy information in decision making. Information Fusion. 2017; 38: 1–11.

[23] Yager RR. The power average operator. IEEE Transactions on Systems, Man and Cybernetics: Part A. 2001; 31(6): 724–731. 10.1109/3477.907562 18244765

[24] Wan SP, Yi ZH. Power average of trapezoidal intuitionistic fuzzy numbers using strict t-norms and t-conorms. IEEE Transactions on Fuzzy Systems. 2016; 24(5): 1035–1047.

[25] Zhang ZM. Hesitant fuzzy power aggregation operators and their application to multiple attribute group decision making. Information Sciences. 2013; 234: 150–181.

[26] Xu YJ, Wang HM. Approaches based on 2-tuple linguistic power aggregation operators for multiple attribute group decision making under linguistic environment. Applied Soft Computing. 2011; 11(5): 3988–3997.

[27] Liang DC, Zhang YRJ, Cao W. q-Rung orthopair fuzzy Choquet integral aggregation and its application in heterogeneous multicriteria two-sided matching decision making. International Journal of Intelligent Systems. 2019; 34(12): 3275–3301.

[28] Rong Y, Liu Y, Pei Z. Interval-valued intuitionistic fuzzy generalised Bonferroni mean operators for multi-attribute decision making. International Journal of Fuzzy Systems. 2021; doi: 10.1007/s40815-021-01064-3

[29] Wei GW, Gao H, Wei Y. Some q-rung orthopair fuzzy Heronian mean operators in multiple attribute decision making. International Journal of Intelligent Systems. 2018; 33(7): 1426–1458.

[30] Wei GW, Lu M. Pythagorean fuzzy Maclaurin symmetric mean operators in multiple attribute decision making. International Journal of Intelligent Systems. 2018; 33(5): 1043–1070.

[31] Liu PD, Teng F. Some Muirhead mean operators for probabilistic linguistic term sets and their applications to multiple attribute decision-making. Applied Soft Computing. 2018; 68: 396–431.

[32] He YD, He Z, Wang GD, Chen HY. Hesitant fuzzy power Bonferroni means and their application to multiple attribute decision making. IEEE Transactions on Fuzzy Systems. 2015; 23(5): 1655–1668.

[33] Liu PD. Multiple attribute group decision making method based on interval-valued intuitionistic fuzzy power Heronian aggregation operators. Computers & Industrial Engineering. 2017; 108: 199–212.

[34] Liu PD, Chen SM, Wang P. Multiple-attribute group decision-making based on q-rung orthopair fuzzy power Maclaurin symmetric mean operators. IEEE Transactions on Systems, Man and Cybernetics: Systems. 2020; 50(10): 3741–3756.

[35] Wang J, Shang XP, Bai KY, Xu Y. A new approach to cubic q-rung orthopair fuzzy multiple attribute group decision-making based on power Muirhead mean. Neural Computing and Applications. 2020; 32(17): 14087–14112.

[36] Iampan A, Garcia GS, Riaz M, Farid HMA, Chinram R. Linear diophantine fuzzy Einstein aggregation operators for multi-criteria decision-making problems. Journal of Mathematics. 2021; 2021: 5548033.

[37] Riaz M, Garg H, Farid HMA, Chinram R. Multi-criteria decision making based on bipolar picture fuzzy operators and new distance measures. CMES-Computer Modeling in Engineering & Sciences. 2021; 127(2): 771–800.

[38] Ramakrishnan KR, Chakraborty S. A cloud TOPSIS model for green supplier selection. Facta Universitatis-Series Mechanical Engineering. 2020; 18(3): 375–397.

[39] Gorsevski PV, Donevska KR, Mitrovski CD, Frizado JP. Integrating multi-criteria evaluation techniques with geographic information systems for landfill site selection: A case study using ordered weighted average. Waste Management. 2012; 32(2): 287–296. doi: 10.1016/j.wasman.2011.09.023 22030279

[40] Kahraman C, Cebi S, Onar SC, Oztaysi B. A novel trapezoidal intuitionistic fuzzy information axiom approach: An application to multicriteria landfill site selection. Engineering Applications of Artificial Intelligence. 2018; 67: 157–172.

[41] Muirhead RF. Some methods applicable to identities and inequalities of symmetric algebraic functions of n letters. Proceedings of the Edinburgh Mathematical Society. 1902; 21: 144–162.

[42] Xiao SJ, Dong HJ, Geng Y, Tian X, Liu C, Li HF. Policy impacts on Municipal Solid Waste management in Shanghai: A system dynamics model analysis. Journal of Cleaner Production. 2020; 262: 121366.

[43] Demesouka OE, Vavatsikos AP, Anagnostopoulos KP. Suitability analysis for siting MSW landfills and its multicriteria spatial decision support system: Method, implementation and case study. Waste Management. 2013; 33(5): 1190–1206. doi: 10.1016/j.wasman.2013.01.030 23453354

[44] De Feo G, De Gisi S, De Vita S, Notarnicola M. Sustainability assessment of alternative end-uses for disused areas based on multi-criteria decision-making method. Science of the Total Environment. 2018; 631–632: 142–152. doi: 10.1016/j.scitotenv.2018.03.016 29524891

[45] Osra FA, Ozcan HK, Alzahrani JS, Alsoufi MS. Municipal Solid Waste characterization and landfill gas generation in Kakia landfill, Makkah. Sustainability. 2021; 13(3): 1462.

[46] Xu Y, Shang XP, Wang J, Wu W, Huang HQ. Some q-rung dual hesitant fuzzy Heronian mean operators with their application to multiple attribute group decision-making. Symmetry-Basel. 2018; 10(10): 472.

[47] Wang J, Wei GW, Wei C, Wei Y. Dual hesitant q-rung orthopair fuzzy Muirhead mean operators in multiple attribute decision making. IEEE Access. 2019; 7: 67139–67166.

[48] Wang J, Wang P, Wei GW, Wei C, Wu J. Some power Heronian mean operators in multiple attribute decision-making based on q-rung orthopair hesitant fuzzy environment. Journal of Experimental & Theoretical Artificial Intelligence. 2020; 32(6): 1–29.

[49] Li L, Lei HG, Wang J. q-Rung probabilistic dual hesitant fuzzy sets and their application in multi-attribute decision-making. Mathematics. 2020; 8(9): 1574.

[50] Ali Z, Mahmood T, Ullah K, Khan Q. Einstein geometric aggregation operators using a novel complex interval-valued pythagorean fuzzy setting with application in green supplier chain management, Reports in Mechanical Engineering. 2021; 2(1): 105–134.

[51] Liu PD, Mahmood T, Ali Z. Complex q-rung orthopair fuzzy aggregation operators and their applications in multi-attribute group decision making. Information. 2020; 11(2): 5.

[52] Riaz M, Hamid MT, Afzal D, Pamucar D, Chu YM. Multi-criteria decision making in robotic agrifarming with q-rung orthopair m-polar fuzzy sets. Plos One. 2021; 16(2): e0246485. doi: 10.1371/journal.pone.0246485 33630877 PMC7906358

[53] Mahmood T, Ullah K, Khan Q, Jan N. An Approach Towards Decision Making and Medical Diagnosis Problems Using the Concept of Spherical Fuzzy Sets. Neural Computing and Applications. 2019; 31: 7041–7053.

